# Unique Spatial Transcriptomic Profiling of the Murine Femoral Fracture Callus: A Preliminary Report

**DOI:** 10.3390/cells13060522

**Published:** 2024-03-16

**Authors:** Will Jiang, Dennis L. Caruana, Jungho Back, Francis Y. Lee

**Affiliations:** Department of Orthopaedics & Rehabilitation, Yale School of Medicine, 47 College Place, New Haven, CT 06510, USA

**Keywords:** spatial transcriptomics, fracture callus, metastatic breast cancer, pathological fracture, interzone

## Abstract

Fracture callus formation is a dynamic stage of bone activity and repair with precise, spatially localized gene expression. Metastatic breast cancer impairs fracture healing by disrupting bone homeostasis and imparting an altered genomic profile. Previous sequencing techniques such as single-cell RNA and in situ hybridization are limited by missing spatial context and low throughput, respectively. We present a preliminary approach using the Visium CytAssist spatial transcriptomics platform to provide the first spatially intact characterization of genetic expression changes within an orthopedic model of impaired fracture healing. Tissue slides prepared from BALB/c mice with or without MDA-MB-231 metastatic breast cancer cells were used. Both unsupervised clustering and histology-based annotations were performed to identify the hard callus, soft callus, and interzone for differential gene expression between the wild-type and pathological fracture model. The spatial transcriptomics platform successfully localized validated genes of the hard (*Dmp1*, *Sost*) and soft callus (*Acan*, *Col2a1*). The fibrous interzone was identified as a region of extensive genomic heterogeneity. MDA-MB-231 samples demonstrated downregulation of the critical bone matrix and structural regulators that may explain the weakened bone structure of pathological fractures. Spatial transcriptomics may represent a valuable tool in orthopedic research by providing temporal and spatial context.

## 1. Introduction

Breast cancer is one of the most common cancers worldwide, with an estimated annual incidence of 2 million [1]. Of those with advanced disease, as many as 75% will develop bone metastases [2]. Bone metastases are frequently osteolytic and predispose patients to a high risk of pathological fracture. Metastatic breast cancer cells accumulate in bone and alter the local skeletal microenvironment via the production of factors such as parathyroid hormone-related protein, hypoxia-inducible factor 1α, and interleukin-6 to facilitate an osteoclastogenic setting [3,4,5]. This directly disrupts normal fracture healing and complicates the clinical management for patients with breast cancer.

Fracture healing contains four distinct stages: an inflammatory reaction with hematoma formation, a bridging soft callus, an ossifying hard callus, and bone remodeling. Surrounding the fracture site is a dynamic zone of recomposition. During normal fracture repair, an intense network of key biological growth factors such as vascular endothelial growth factor, transforming growth factor β, and bone morphogenetic proteins precisely regulate the transition between fracture healing stages [6]. The presence of metastatic breast cancer cells in the skeletal compartment impairs osteoblastic anabolism and drives osteoclastogenic characteristics, such as through activation of the pERK1/2 pathway [7]. Given that fracture healing progresses through distinctive phases and zones of activity, a spatial and temporal understanding is required to better understand the imbalances of osteoblast and osteoclast activity that result in impaired fracture healing states.

Previous efforts to characterize the fracture callus have relied on methods (such as single-cell RNA, in situ hybridization, cDNA arrays) that either remove spatial context or rely on the targeted selection of genes of interest [8,9,10,11]. These approaches may be inadequate in capturing the diverse, spatially localized expression profile of a healing fracture callus. The recent emergence of spatial transcriptomic technology has finally enabled the mapping of the entire transcriptome without loss of morphological context [12]. Applications have included areas such as tumor studies, brain mapping, lung diseases, and kidney disease [13]. To our knowledge, no prior study has applied this technology in orthopedic research.

In this pilot investigation, we apply a popular spatial transcriptomics platform to analyze a region of bone known to exhibit high spatial variability in gene expression. We use a validated pathological fracture model to demonstrate gene expression changes across distinctive regions of the fracture callus. We aim to describe the methodological process of selecting a region of interest, comparing relevant zones of interest, and using downstream bioinformatics softwareto preliminarily identify up- and down-regulated genes.

## 2. Materials and Methods

### 2.1. Cell Selection and Preparation

MDA-MB-231 (*Homo sapiens*) triple-negative breast adenocarcinoma cells were used. A human cell line was selected to ensure the spatial transcriptomics platform would not detect the expression of breast cancer cells. This allows for detection of only mouse femur-specific gene changes. The MDA-MB-231 cells originated from the American Type Culture Center (ATCC, Manassas, VA, USA). Cells were cultured in DMEM supplemented with 10% FBS, 100 IU·mL^−1^ penicillin and 100 μg·mL^−1^ streptomycin (Thermo Fisher Scientific, Inc., Waltham, MA, USA).

### 2.2. In Vivo Pathological Fracture Model

BALB/C mice aged 16–18 weeks were purchased from Charles River Laboratories (Wilmington, MA, USA). A femoral osteotomy was performed. Ketamine (10 mg/mL) and xylazine (1 mg/mL) were used as anesthesia agents. A Gigli saw (0.22 mm diameter) was used to produce a transverse midshaft femoral osteotomy. The wild-type control was injected with vehicle only while the pathological fracture healing mice were given an intramedullary injection of 0.5 × 10^6^ cells of MDA-MB-231. For the pathological fracture model, mice were sacrificed at 1 week and 2 weeks post-operative. The wild-type mouse was sacrificed at 2 weeks post-osteotomy. This study was approved by the Yale University Institutional Animal Care & Use Committee.

### 2.3. Tissue Harvest and Histology Preparation

Femurs were harvested upon sacrifice and immediately fixed in a solution of 4% paraformaldehyde and PBS. Decalcification was performed with 10% EDTA (pH 7.2–7.4) for 2 weeks on a shaker and embedded in paraffin. Paraffin blocks were sectioned at 5 μm and baked in an oven at 60 °C for 20 min before hematoxylin and eosin (H&E) staining. RNA curls off the paraffin block were collected and sent for verification of RNA quality. All blocks met the DV200 criteria for formaldehyde fixed paraffin embedded samples of at least 30% (wild type: 42%, 1-week MDA-231: 36%, 2-week MDA-231: 40%). Samples were then processed in accordance with the Visium CytAssist protocol (10× Genomics, Pleasanton, CA, USA) using the Mouse Probe Set v1.

### 2.4. Visium Data Processing and Analysis

**Figure 1:**
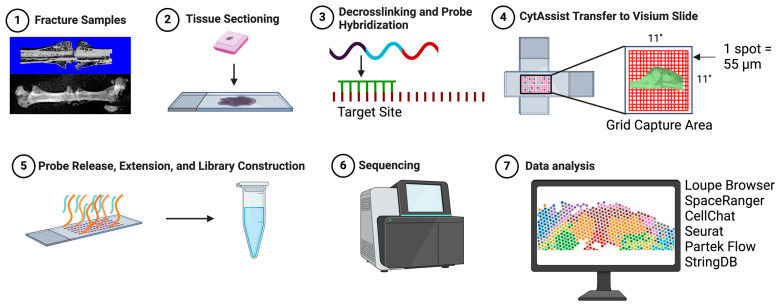
Overview of 10× Genomics CytAssist Visium spatial transcriptomics platform. (1) Mouse femurs are harvested at one or two weeks. (2) Femur is fixed with 4% paraformaldehyde and embedded in paraffin. Tissue is sectioned at 5 μm. (3) Decrosslinking and probe hybridization are performed. (4) The user supplied slide is then aligned with a Visium CytAssist slide using an 11″ by 11″ capture area for sequencing. The capture area contains 14,000 tissue capture spots (each spot is 55 μm in diameter). (5) Attached probes are released with RNAse allowing for probe extension and subsequent library construction. Each probe is now attached with a barcoded label with spatial information. (6) Sequencing is performed. (7) The final step is data analysis by the user. Popular bioinformatics options include Loupe Browser (10× Genomics), SpaceRanger (10× Genomics), CellChat, Seurat, Partek Flow, and StringDB. Visualization created by BioRender.

A total of three samples were processed (Figure 1). Visium Spatial Gene Expression Slides (11″ by 11″ capture area) contain 14,000 gene detection spots, each 55 μm in diameter. Read alignment and immediate raw data processing was performed by an experienced bioinformatician at the University’s Genome Center using Space Ranger (v2.0.1; 10× Genomics). A filtered feature matrix (h5) for each sample was imported into Partek Flow (v11.0, Chesterfield, MO, USA). First, all gene detection spots in the entire fracture callus were selected based on histology. A feature count filter was applied to exclude features with less than 1.0 in at least 80% of cells. SCTransform (Seurat) was performed for normalization [14]. Unsupervised graph-based clustering was performed based on a Louvain clustering algorithm with a resolution of 0.5 after dimensionality reduction with principal component analysis. Biomarkers were then computed for each cluster with a positive fold change threshold of 1.5. Second, the hard callus, soft callus, and interzone were identified on the two-week wild-type and two-week MDA-231. An early interzone was identified on the one-week MDA-231—at one week, the fracture callus has not yet fully formed. Cells with counts greater than 1 and features greater than 100 were included. Data were normalized via SCTransform (Seurat). Respective regions were compared using ANOVA and a differential expression gene list was generated. Downstream analysis consisted of unweighted and weighted analysis. For unweighted analysis, a filtered list of *p*-value less than 0.05, false discovery rate (FDR) less than 0.01, and fold-change of at least −2 or +2 was generated. A list of gene names was then entered into StringDB (v12.0). For weighted analysis, the filtered gene list with expression data was imported into Ingenuity Pathway Analysis (QIAGEN, v23.0).

Due to mixed cell lineages within each callus area and the tissue detection size of 55 μm, cell lineage-based analysis (i.e., osteoblasts, chondrocytes, and osteoclasts) could not be accurately performed. This spatial transcriptomics platform does not have a single-cell resolution.

## 3. Results

A total of three samples were included, representing two distinct time points (one and two weeks) of the pathological fracture MDA-MB-231 callus and a two-week wild-type control callus. A total of 17,612 cells and 19,454 features were analyzed. Data quality is available in Supplementary Table S1.

### 3.1. Validation of Spatial Transcriptomics via Identification of Known Marker Genes

**Figure 2:**
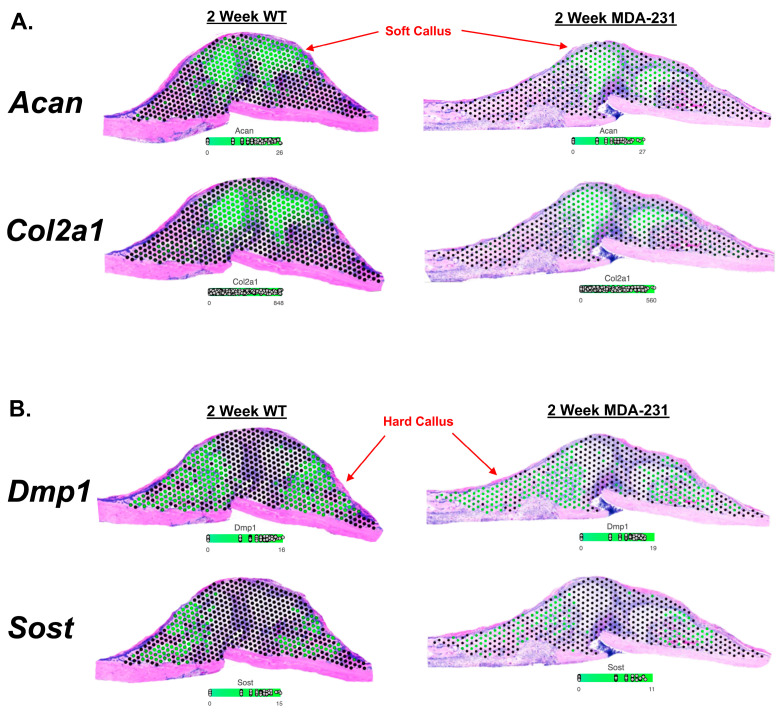
Visium-based spatial localization of known genetic markers of the soft and hard callus. Known genetic markers of the soft and hard callus were used to support validation of the Visium spatial transcriptomics platform. Green dots represent areas of gene expression of a particular gene. All expression levels are log2-transformed raw expression counts. (**A**) Aggrecan (*Acan*) and Type II Collagen (*Col2a1*) are known to be localized to the soft callus. Spatial transcriptomics shows predominant localization in the soft callus. (**B**) Dentin matrix acidic phosphoprotein 1 (*DMP1*) and Sclerostin (*Sost*) are known to be localized to the hard callus. Spatial transcriptomics shows predominant localization in the hard callus.

Known markers were used to determine whether the spatial transcriptomics platform could correctly localize gene expression (Figure 2). *Acan* is a highly specific chondrocyte marker localized to the soft callus that was used to preliminarily validate spatial localization of the platform [15]. Similarly, *Col2a1*, a highly expressed marker of proliferative chondrocytes, was also well-localized to the soft callus as expected [16]. Dentin matrix protein 1 (*DMP1*) is a known non-collagenous matrix protein expressed in the maturation of osteoblasts and involved in callus mineralization with high localization to the hard callus [17,18]. The regulator, sclerostin (*Sost*), is also well-localized to the hard callus as an inhibitive regulator of bone formation [19]. Both *DMP1* and *Sost* were found to be highly expressed in the hard callus with minimal to no detection in the soft callus or interzone (Figure 2).

### 3.2. Unsupervised Global Gene Clustering

**Figure 3:**
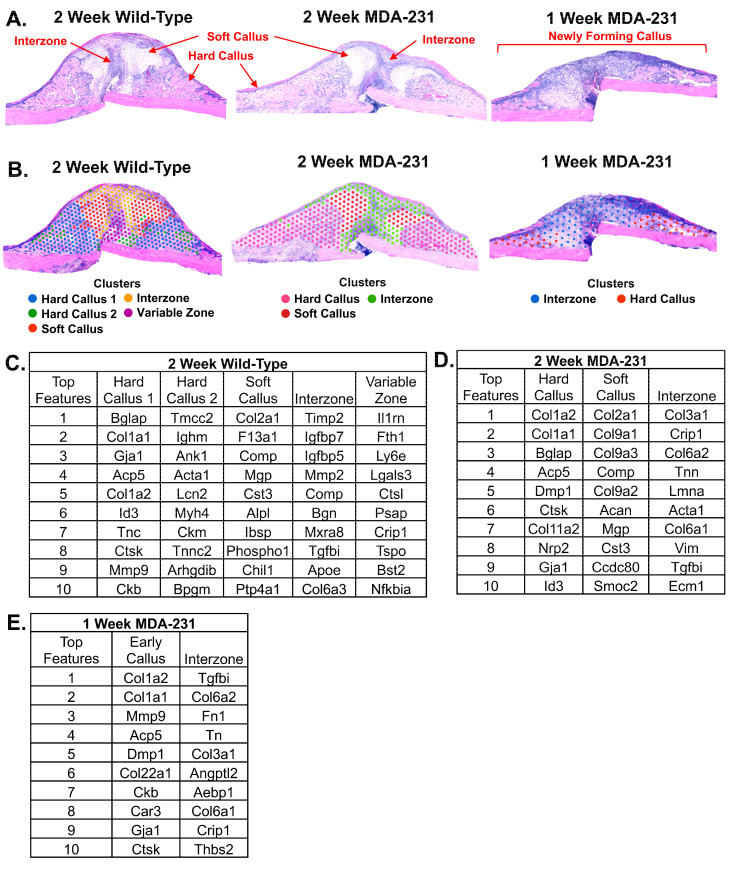
Unsupervised analysis of fracture callus. (**A**) H&E images of fracture callus histology. Wild-type and MDA-MB-231 (pathological fracture model) fracture calluses are shown at two weeks post-femur fracture. A one-week MDA-MB-231 fracture callus is also shown. (**B**) Unsupervised software-generated graph-based clustering (0.50 resolution) is overlayed on H&E images (Partek Flow, v11.0, Chesterfield, MO, USA). Two-week wild-type callus reveals five distinct clusters that histologically correspond to hard callus (two clusters), soft callus, interzone, and a variable section of the interzone. Two-week MDA-231 shows three zones (hard callus, soft callus, and interzone). One-week MDA-231 identified two zones corresponding to a preliminary interzone and hard callus. At one week, the fracture callus has not yet fully developed. (**C**–**E**) Top 10 genes expressed within each cluster (positive fold change threshold at 1.5).

In both two-week samples (MDA-231 and wild-type), unsupervised graph-based clustering aligned with histological identification of the hard callus, soft callus, and interzone (Figure 3A,B).

Five independent clusters were identified in the two-week wild-type fracture callus (Figure 3C). The primary hard callus cluster included expression of the osteoblastic marker osteocalcin (*Bglap*) and Type I collagen (*Col1a1*). A small subset of the hard callus that localized to tissue spots adjacent to the surrounding muscle expressed more muscle-related markers such as actin alpha-1 (*Acta1*), myosin heavy chain 4 (*Myh4*), creatine kinase (*Ckm*), and the skeletal muscle marker troponin C2 (*Tnnc2*). In the soft callus cluster of the wild-type mouse, the highest expressed gene was *Col2a1*, reflective of the chondrocyte cell population. Another chondrocyte identifier, *Comp*, was the third most highly expressed gene according to unsupervised graph-based clustering of the soft callus. A novel, highly heterogenous zone within the interzone abutting the fracture line was identified. Genes expressed in this variable zone included *Il1rn*, *Fth1*, *Ly6e*, *Lgals3*, *Ctsl*, *Psap*, *Crip1*, *Tspo*, *Bst2*, and *Nfkbia*. The full dataset is available in Supplementary Table S2.

In the two-week callus of MDA-MB-231, three clusters were generated and representative of the hard callus, soft callus, and interzone (Figure 3D). The hard callus also exhibited high expression of Type 1 collagen (*Col1a2* and *Col1a1*). *Bglap* (osteocalcin) was also highly expressed in the hard callus, reflective of a non-collagenous protein that is highly abundant in bone matrix [20]. The soft callus expressed chondrocyte markers such as *Col2a1*, *Comp*, and aggrecan (*Acan*). Hyaline-associated cartilage (*Col9a1*, *Col9a2*, *Col9a3*) was also highly expressed in this cluster. The interzone showed high heterogeneity, expressing a mixture of bone matrix, structural, and signaling regulators including *Col3a1*, *Crip1*, *Col6a2*, *Tnn*, *Lmna*, *Acta1*, *Col6a1*, *Vim*, *Tgfbi*, and *Ecm1*. The full dataset is available in Supplementary Table S3.

Two clusters were identified within the fracture callus at one week of the MDA-MB-231 sample (Figure 3E). At one week, the fracture callus has not reached maturity and presents as an amorphous region. Thus, the two clusters were classified as an early callus and a preliminary interzone. The early callus cluster showed high expression of Type 1 collagen (*Col1a2* and *Col1a1*) formation along with *Dmp1*. Other highly expressed genes of the region included *Mmp9*, *Acp5*, *Col22a1*, *Ckb*, *Car3*, *Gja1*, and *Ctsk*. The preliminary interzone displayed large heterogeneity and diversity in gene expression (*Tgfbi*, *Col6a2*, *Fn1*, *Tn*, *Col3a1*, *Angptl2*, *Aebp1*, *Col6a1*, and *Thbs2*). The full dataset is available in Supplementary Table S4.

### 3.3. Comparison of Gene Expression between Two-Week MDA-MB-231 and Wild-Type Fracture Callus

**Figure 4:**
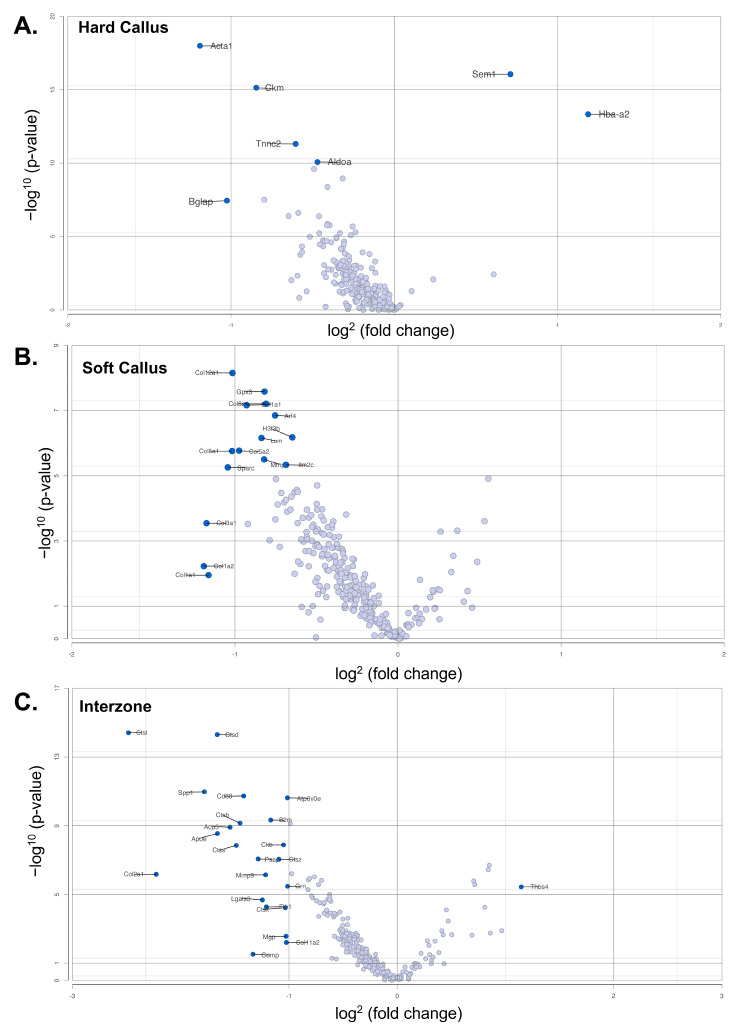
Volcano plot of differential gene expression between two-week pathological fracture model (MDA-231) and two-week wild-type control. A total of 289 genes compared using ANOVA. Each dot represents a single detected gene. Genes meeting the *p*-value and fold change cutoffs (FC at least 1.0, *p*-value < 0.05) are colored in blue and labeled with their corresponding gene names. Lightly shaded blue dots without labels did not reach the level of statistical significance. (**A**) Differential expression comparison of the hard callus. (**B**) Differential expression comparison of the soft callus. (**C**) Differential expression comparison of the interzone.

H&E images were used to identify probe spots in the hard callus, soft callus, and interzone of the two-week MDA-MB-231 and wild-type calluses. After filtering and normalization, a total of 289 features were available for comparison between the three regions of interest (Figure 4).

**Figure 5:**
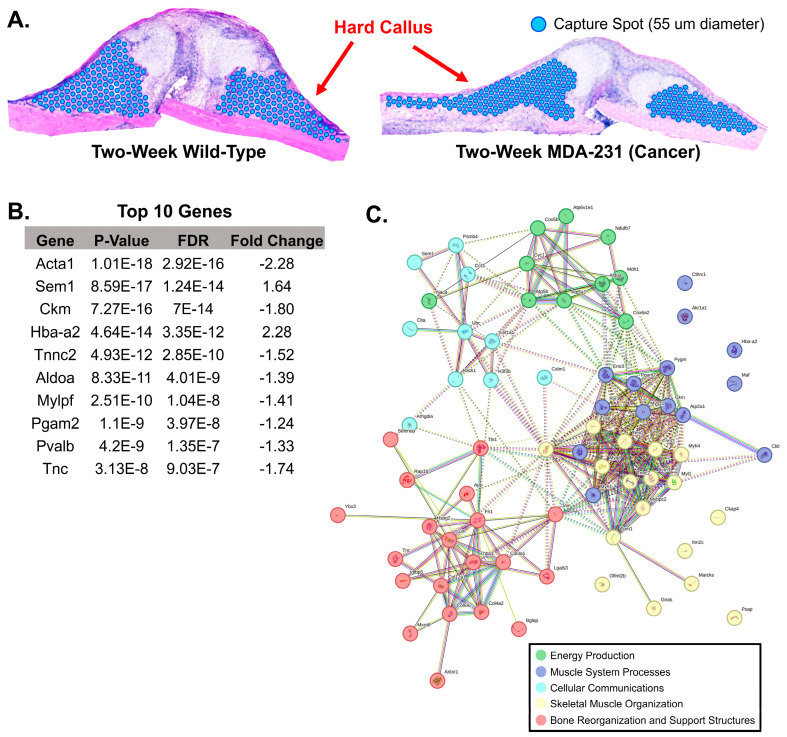
Comparison of hard callus gene expression between two-week wild-type and two-week MDA-231. (**A**) Using the H&E image, the hard callus on each sample was identified manually and all corresponding gene expression capture spots were selected. Blue dots represent selected gene expression capture spots included in the analysis. (**B**) A table of the top 10 genes ANOVA differential expression revealed 71 differentially expressed genes (*p*-value < 0.05, false discovery rate < 0.01, fold change −1 to 1) between the two-week wild-type and two-week MDA-231. (**C**) Cluster-based visualization of all 71 differentially expressed genes. Top associated functions include energy production, muscle system processes, cellular communications, skeletal muscle organization, and bone reorganization and support structures (StringDB (v12.0), Protein–Protein Interaction Enrichment *p*-value < 0.0001 for network analysis).

In the hard callus, a total of 499 cells were included in analysis after filtering and normalization (Figure 5A). ANOVA analysis identified 71 genes (69 downregulated; 2 upregulated) that were differentially expressed across the hard callus of the two samples (*p* < 0.05, false discovery rate (FDR) < 0.01, fold change at least −1 to 1). *Sem1*, a conserved subunit of the mammalian proteasome including ties to BRCA2 stabilization, was found to be upregulated (*p* < 0.0001, FDR < 0.0001, fold change: 1.64). The vast majority of differentially expressed genes demonstrated significant downregulation in the hard callus of the two-week MDA-MB-231 pathological fracture callus (Figure 5B). This included key expressed genes of the wild-type fracture hard callus, such as *Tnc* and *Bglap*. StringDB identified five distinct categorizations of the differentially expressed genes including roles in energy production, muscle system processes, cellular communications, skeletal muscle organization, and bone reorganization and support structures (Figure 5C). Stricter filtering of fold change (from −2 to 2) identified three differentially expressed genes (*Bglap* (fold change: −2.04)), *Acta1* (fold change: −2.28, *Hba-a2* (fold change: 2.28)). A full list of differentially expressed genes is available in Supplementary Table S5.

**Figure 6:**
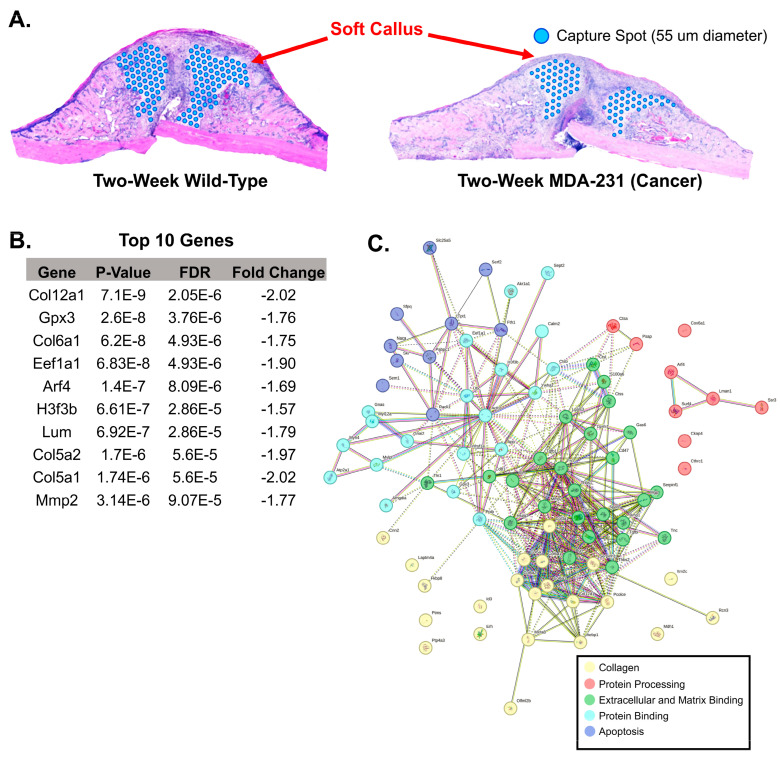
Comparison of soft callus gene expression between two-week wild-type and two-week MDA-231. (**A**) Using the H&E image, the soft callus on each sample was identified manually and all corresponding gene expression capture spots were selected. Blue dots represent selected gene expression capture spots included in the analysis. (**B**) A table of the top 10 genes ANOVA differential expression revealed 86 differentially expressed genes (*p*-value < 0.05, false discovery rate < 0.01, fold change −1 to 1) between the two-week wild-type and two-week MDA-231. (**C**) Cluster-based visualization of all 86 differentially expressed genes. Top associated functions include collagen, protein processing, extracellular and matrix binding, protein binding, and apoptosis (StringDB (v12.0), Protein–Protein Interaction Enrichment *p*-value < 0.0001 for network analysis).

Analysis of the soft callus included 230 cells (Figure 6A). A total of 86 genes were found to be differentially expressed between the soft calluses of the MDA-MB-231 sample and the wild-type sample (*p* < 0.05, FDR < 0.01, fold change at least −1 to 1). Similar to the hard callus, differential expression analysis primarily showed down-regulation in the MDA-MB-231 (81 downregulated, 5 upregulated) (Figure 6B). The soft callus of the MDA-MB-231 sample similarly exhibited upregulation of *Sem1* as seen in the hard callus comparison. The matrix protein (*Mgp*) was also increased in the MDA-MB-231 sample. Two myosin-associated genes were also found to be upregulated in the pathological fracture sample (*Myh4* and *Mylpf*). *SPARC*, a key glycoprotein involved in Type I collagen binding and extracellular matrix stabilization, was downregulated in the MDA-MB-231 sample [21]. StringDB analysis indicated four biological domains of function represented by the differentially expressed genes: collagen, protein processing, extracellular and matrix binding, and protein binding (Figure 6C). The four genes with a fold change of at least −2 to 2 were *Col12a1*, *Col5a1*, *Sparc*, and *Col3a1*. Supplementary Table S6 provides a comprehensive list of all differentially expressed genes.

**Figure 7:**
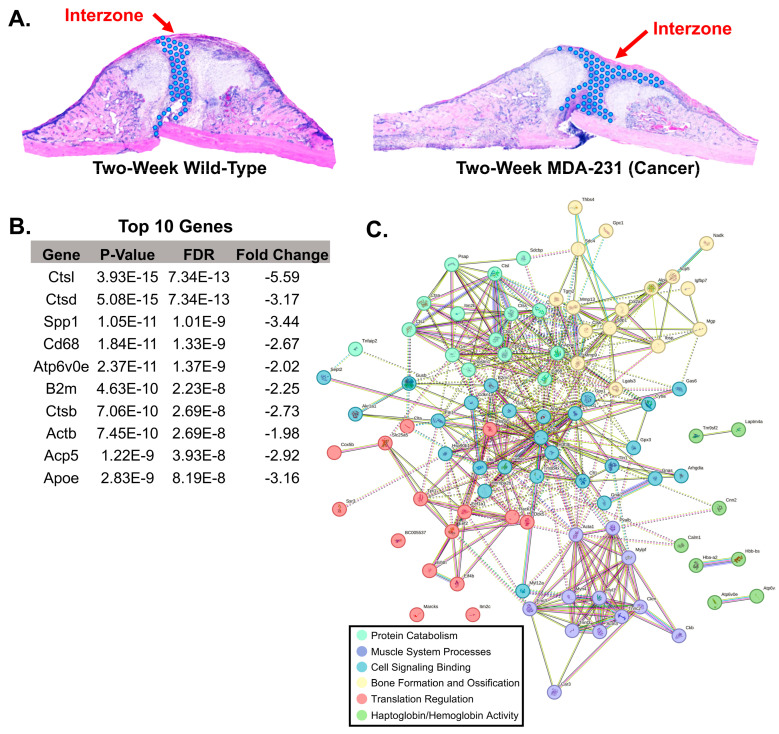
Comparison of interzone gene expression between two-week wild-type and two-week MDA-231. (**A**) Using the H&E image, the interzone on each sample was identified manually and all corresponding gene expression capture spots were selected. Blue dots represent selected gene expression capture spots included in the analysis. (**B**) A table of the top 10 genes ANOVA differential expression revealed 92 differentially expressed genes (*p*-value < 0.05, false discovery rate < 0.01, fold change −1 to 1) between the two-week wild-type and two-week MDA-231. (**C**) Cluster-based visualization of all 92 differentially expressed genes. Top associated functions include protein catabolism, muscle system processes, cell signaling binding, bone formation and ossification, translation regulation, and haptoglobin/hemoglobin activity (StringDB (v12.0), Protein–Protein Interaction Enrichment *p*-value < 0.0001 for network analysis).

The comparison of the interzones included 121 cells (Figure 7A). A total of 92 differentially expressed genes (*p* < 0.05, FDR < 0.01, fold change at least −1 to 1) were identified, affecting protein catabolism, muscle system processes, cell signaling binding, bone formation and ossification, translation regulation, and haptoglobin/hemoglobin activity (StringDB) (Figure 7B,C). A total of 14 genes were identified as upregulated (*Thbs4*, *Hbb-bs*, *Hba-a2*, *Tnnc2*, *Mylpf*, *Myh4*, *Tnnt3*, *Acta1*, *Car3*, *Eno3*, *Myl1*, *Ckm*, *Actn3*, *Pvalb*) within the MDA-MB-231 interzone and 78 genes were found to be downregulated (complete list found in Supplementary Table S7). The greatest heterogeneity between the wild-type and MDA-231 sample was identified within the interzone. A total of 21 genes were identified based on stricter fold change criteria (from −2 to 2) including: *Ctsl*, *Ctsd*, *Spp1*, *Cd68*, *Atp6v0e*, *B2m*, *Ctsb*, *Acp5*, *Apoe*, *Ckb*, *Ctss*, *Psap*, *Ctsz*, *Col2a1*, *Mmp9*, *Grn*, *Thbs4*, *Lgals3*, *Fth1*, *Ctsk*, and *Mgp.*

### 3.4. Pathway Analysis: Comparison of Two-Week MDA-MB-231 and Wild-Type Fracture Callus

Pathway analysis (Ingenuity Pathway Analysis, QIAGEN, v23.01) was performed for the differentially expressed genes between the MDA-MB-231 and wild-type hard callus, soft callus, and interzone.

**Figure 8:**
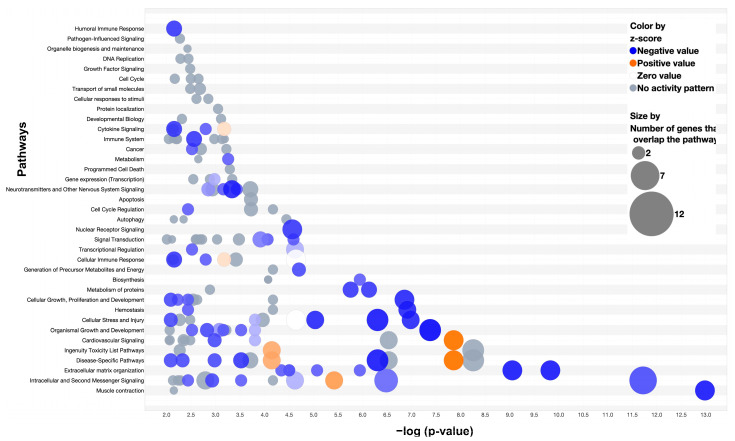
Pathway Analysis of Differentially Expressed Genes of the Hard Callus (2-Week Wild-type vs. MDA-231). A bubble plot of significant pathways (*p*-value < 0.05, QIAGEN Ingenuity Pathway Analysis v01-22-01) shared by 71 differentially expressed genes between the two-week wild-type and the MDA-231 (*p*-value < 0.05, false discovery rate < 0.01, fold change −1 to 1). Blue bubbles indicate decreased gene expression of the MDA-231 compared to wild-type. Larger bubbles indicate more genes that overlap with the pathway. Shading reflects z-score values (darker = larger z-scores).

Top canonical pathways of the hard callus differential expression included striated muscle contraction, calcium signaling, integrin cell surface interactions, and extracellular matrix organization (all *p* values < 0.0001) (Figure 8). Pathway analysis identified cancer (67 genes), organismal injury and abnormalities (69 genes), and skeletal and muscular disorders (54 genes) as the three diseases and disorders with the greatest number of genes involved from our hard callus differential expression gene list (all *p* values < 0.0001). Molecular and cellular function pathways identified included cell morphology, cellular movement, cell-to-cell signaling and interaction, cell death and survival, and cellular assembly and organization (all *p* values < 0.0001).

**Figure 9:**
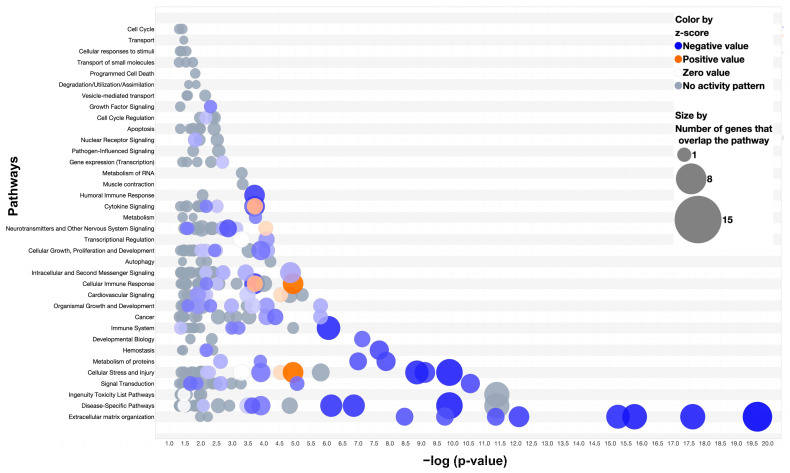
Pathway Analysis of Differentially Expressed Genes of the Soft Callus (2-Week Wild-type vs. MDA-231). A bubble plot of significant pathways (*p*-value < 0.05, QIAGEN Ingenuity Pathway Analysis v01-22-01) shared by 86 differentially expressed genes between the two-week wild-type and the MDA-231 (*p*-value < 0.05, false discovery rate < 0.01, fold change −1 to 1). Blue bubbles indicate decreased gene expression of the MDA-231 compared to wild-type. Larger bubbles indicate more genes that overlap with the pathway. Shading reflects z-score values (darker = larger z-scores).

Analysis of the soft callus genes identified the top canonical pathways as extracellular matrix organization, assembly of collagen fibrils and other multimeric structures, integrin cell surface interactions, collagen biosynthesis and modifying enzymes, and collagen degradation (all *p* values < 0.0001) (Figure 9). The three diseases and disorders with the greatest number of gene involvement included cancer (83 genes), organismal injury and abnormalities (83 genes), and connective tissue disorders (53 genes) (all *p* values < 0.0001). Cancer-associated upstream regulators identified through pathway analysis included *CCR2* (inhibition), *TGFBI* (inhibition), and *TP53* (inhibition), *p* values < 0.0001.

**Figure 10:**
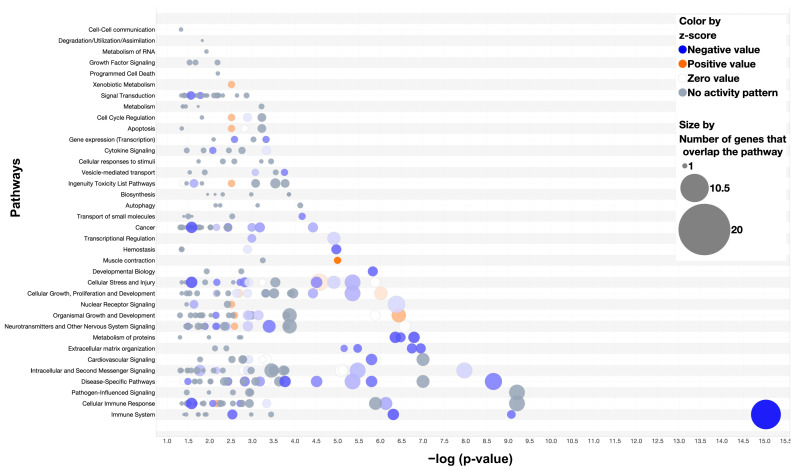
Pathway Analysis of Differentially Expressed Genes of the Interzone (2-Week Wild-type vs. MDA-231). A bubble plot of significant pathways (*p*-value < 0.05, QIAGEN Ingenuity Pathway Analysis v01-22-01) shared by 92 differentially expressed genes between the two-week wild-type and the MDA-231 (*p*-value < 0.05, false discovery rate < 0.01, fold change −1 to 1). Blue bubbles indicate decreased gene expression of the MDA-231 compared to wild-type. Larger bubbles indicate more genes that overlap with the pathway. Shading reflects z-score values (darker = larger z-scores).

Within the interzone, significant canonical pathways included neutrophil degranulation, phagosome maturation, trafficking and processing of endosomal TLR, and RhoGDI signaling, *p* < 0.0001 for all (Figure 10). Relevant disease and disorder pathways identified included organismal injury and abnormalities (89 genes), skeletal and muscular disorders (73 genes), and inflammatory response (65 genes), *p* values < 0.0001. Upstream regulators of interest included cancer-associated regulators such as *TGFBI* (inhibition) and *TP53* (inhibition) alongside the wound-healing regulator *FN1* (inhibition), *p* values < 0.0001.

### 3.5. Comparison of One-Week and Two-Week MDA-MB-231 Interzone

**Figure 11:**
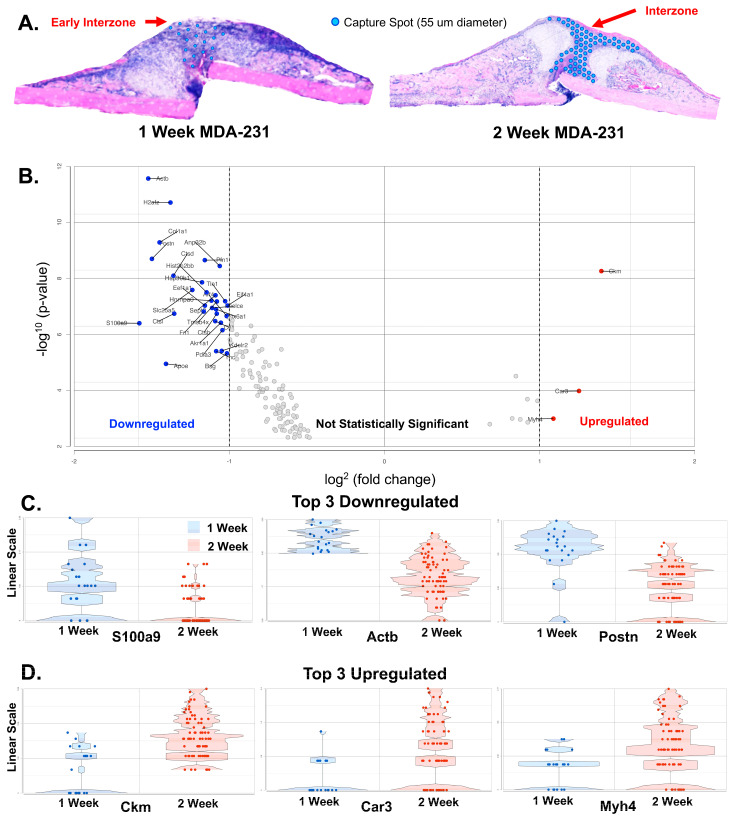
Differential expression between 1-week and 2-week MDA-231 interzone. (**A**) Hematoxylin and eosin image of 1-week MDA-231 and 2-week MDA-231. Blue circles represent the individual capture spots selected to represent the preliminary interzone of the 1-week MDA-231 fracture callus and the fully formed interzone of the 2-week MDA-231 callus. (**B**) Volcano plot demonstrating the distribution of up-regulated and down-regulated genes in reference to the 1-week MDA231 interzone. Labeled red dots are genes that are upregulated while labeled blue dots are downregulated genes. ANOVA analysis revealed 33 differentially expressed genes (*p*-value < 0.05, false discovery rate < 0.01, fold change −1 to 1). (**C**) Violin plots of the top 3 (fold-change) downregulated genes. X-axis is the sample type and the y-axis is the raw expression counts (linear scale). (**D**) Violin plots of the top 3 (fold-change) upregulated genes. The X-axis is the sample type and the Y-axis is the raw expression counts (linear scale).

Based on histology, a preliminary region was identified as the preliminary interzone on the one-week MDA-MB-231 sample and compared to the two-week MDA-MB-231 fracture interzone (Figure 11A). A total of 92 cells across the same 289 features as used earlier was included in the comparison. ANOVA analysis identified 145 differentially expressed genes (*p* < 0.05, FDR < 0.01, fold change at least −1 to 1). A total of 33 genes had both a *p* < 0.0001 and fold-change at least −1 to 1 (Figure 11B). The one-week interzone displayed higher expression genes reflective of early wound healing such as *Actb*, *Postn*, *Fn1*, and *Tnc* (Figure 11C). The second-week MDA-MB-231 interzone reflected high expression of genes such as *Ckm*, *Myh4*, *Tnnt3*, *Eno3*, *Tnnc2*, *Actn3*, *Pvalb*, and *Atp2a1* that are involved in muscle function (Figure 11D).

## 4. Discussion

Spatial transcriptomics uniquely characterizes genomic expression with preserved spatial context. The fracture callus is a dynamic zone of cellular activity that possesses complex genomic architecture. In this study, we present a preliminary application of this novel technique to analyze the fracture callus in pathological fracture murine models to assess the viability for use in future orthopedic research.

The spatial context in the fracture is critical. The fracture environment consists of distinctive regions representing the hard callus, soft callus, and fibrous interzone that are dominated by various cell populations including osteoblasts, osteoclasts, chondrocytes, inflammatory cells, endothelial cells, periosteal progenitor cells, and mesenchymal stem cells [22]. The study of this region requires incorporation of spatial context. Khan et al. applied DNA microarray to classify normal fracture healing at multiple time points, although fracture callus and hematoma were extracted and blended together for the RNA sample collection, which removed architectural classification of the callus [8]. The hard and soft callus are the most studied compared to the fibrous interzone, which has seen limited reporting [23].

### 4.1. Pathological Fracture Model: Cell Line Selection

MDA-MB-231 is a well-validated epithelial cell lineage derived from Homo sapiens breast adenocarcinoma that is triple hormone receptor-negative and highly metastatic [24]. Cells of this lineage are aggressive and show poor differentiation. Bone metastases involving MDA-MB-231 have several characteristic findings. Kang et al. identified the unique molecular signature of MDA-MB-231 bone metastasis to feature activation of five key molecular markers in the bone microenvironment: *CXCR4*, *MMP1*, *CTGF*, *FGF5*, and *IL11* [25]. This cell lineage disrupts the physiologic expression of osteoblasts and osteoclasts, favoring osteoclastogenesis. In vitro studies with co-cultured osteoclasts reveal MDA-MB-231 induces osteoclastogenesis and bone resorption via induction of IL-6 [26]. Overlayed on osteoblasts, MDA-MB-231 increases the activity of matrix metalloproteinases to promote tumor metastases and induces osteoblast changes in response to key factors such as *PDGF-C*, *SAA3*, and *OPG*. The presence of metastatic breast cancer induces specific alterations in isolated osteoblasts and osteoclasts, but the timeline of these changes and its spatial localization to the fracture callus is poorly understood.

Visium spatial transcriptomics platform (Mouse Probe Kit v1) is highly selective for mouse transcripts. As a result, a human cell line was selected to avoid detecting cancer cell gene expression. This allows for the interpretation of gene expression changes in the mouse fracture microenvironment only.

### 4.2. Unsupervised Clustering Mirrors Fracture Callus Histology: Predictable Genes and Novel Insights into the Interzone

Clustering analysis of genes showed clusters aligned well with histology. In the two-week wild-type fracture sample, the hard callus cluster expressed predictable genes such as the matrix protein *Bglap* and Type I collagen, both localized to mineralized bone [27]. Type XI collagen, a fibrillary collagen associated with Type I collagen fibrillogenic, was also expressed in the hard callus [28]. *Mmp9* also showed exclusive expression in the hard callus, which matched reports that *Mmp9* deficient mice showed abnormal pathology in hard callus fracture activity [29]. A total of 84 genes on biomarker analysis were identified to be highly associated with hard callus expression, including a mixture of well-characterized genes and lesser-known genes.

The interzone was of great interest due to its highly heterogenous composition. The interzone is difficult to study given its small area, high heterogeneity, and temporal patterns surpass the capabilities of non-spatial transcriptomic techniques. In this study, two clusters were represented by the bridging zone between the two adjacent pieces of the soft callus: a fibrous interzone and a vascularized inflammatory region. An early hypothesis suggests that the fibrous interzone is a dynamic region of regulation and active gene transcription situated in the heart of the fracture site. *Timp2*, part of the tissue inhibitors of metalloproteinase activity, is a regulator of cytokine and chemokine activity that was found to be highly expressed in the interzone [30]. Growth factors such as *Igfbp5* and *Igfbp7* were also highly expressed in the interzone. *Comp*, a chondrocyte marker, showed expression in the interzone, which may be evidence of early soft callus formation taking place at the periphery of the interzone. However, inclusion of tissue spots at the periphery of the interzone-soft callus intersect may account for some mixed gene expression inclusion in clustering. The collagen binding regulator, *Tgfbi*, also demonstrated high expression within the interzone [31]. The interzone also included the expression of inflammation-related genes such as *Lsp1*, *Ski*, *Clu*, and *Cr.* A total of 139 genes were identified as potential biomarkers (*p* < 0.05) within the interzone. The highly vascularized variable zone demonstrated even greater diversity. The interzone appears to be a dynamic, highly heterogenous region of regulation that may govern both the framework of callus formation and inflammatory signaling. Interestingly, directly underneath the interzone is a region with even greater diversity (variable zone). Biomarker analysis revealed 298 genes associated with this region that vastly outnumbered even the genes identified within the interzone (139), *p* < 0.05. Expression within this region directly adjacent to the fracture site appears highly variable. The most significant gene was *Il-1-rn*. Antagonism of the Il-1 receptor may serve anti-Il-1 pro-inflammatory activity to attenuate inflammation at the fracture site [32]. The location of this region directly adjacent to the cortical bone, interzone, and soft callus may allow this zone to play a fundamental role in regulating the callus formation and complex mechanism of fracture repair.

### 4.3. Using Spatial Transcriptomics for Region of Interest Comparisons

The application of this spatial transcriptomic platform also allows for manual selection of regions of interest for differential gene expression. This technique was used to compare the hard callus, soft callus, and interzone of the two-week MDA-MB-231 sample and wild-type control. Due to the timeline of callus formation, the one-week MDA-MB-231 callus was excluded in this part of the analysis due to a lack of identifiable hard callus, soft callus, and interzone regions. Of note, all three regions demonstrated predominant downregulation of genes in the MDA-MB-231 fracture callus compared to the wild-type control. The altered expression profile of pathological fracture models may partially address the reason for failed clinical bone healing in pathological fracture patients [33]. For instance, within the hard callus, we saw evidence of decreased *Bglap* in the MDA-MB-231 sample, which is an important osteoblast secreted protein that mediates hard callus bone remodeling. Other matrix proteins and structural molecules with decreased expression in the MDA-MB-231 hard callus included *Tnc*, *thrombospondin 1* (*Thbs1*), *Cthrc1*, *Col12a1*, and *Col4a2*, amongst others [34,35]. Decreased gene expression of these structural molecules may contribute to weakened structural support of bone in metastatic skeletal disease [36]. While two genes were found to be upregulated in the MDA-MB-231 hard callus (*Hba-a2*, *Sem1*), the biological significance of this regarding fracture healing is unclear.

In comparison to the soft callus, there was significant downregulation of collagen gene expression (*Col3a1*, *Col5a1*, *Col5a2*, *Col6a1*, *Col6a2*, *Col6a3*, *Col12a1*), genes that are involved in fibrillogenic activity and strengthening collagen fibril integrity [37]. Importantly, osteonectin (*Sparc*), which is a major regulator of extracellular matrix assembly and osteoblast/osteoclast activities, was found to be downregulated in the MDA-MB-231 sample [38]. Although differential expression analysis showed four upregulated genes in the MDA-MB-231 soft callus (*Myh4*, *Mylpf*, *Atp2a1*, *Sem1*), the predominance of the muscle-associated protein expression may be a result of detection spot overlap with surrounding muscle tissue in selection of the soft callus.

Analysis of the differential gene expression list of the interzone indicated marked downregulation within the MDA-MB-231 interzone. The most downregulated gene of the MDA-MB-231 interzone, cathepsin L (*Ctsl*), appears to play an important role in bone remodeling, where knockout studies showed decreased trabecular bone formation [39]. *CD68*, an osteoclast-specific gene, displayed downregulation in the MDA-MB-231 interzone. Gene studies of *CD68* suggest that downregulation in osteoclasts leads to inefficient bone resorption and dysfunctional osteoclasts [40]. Downregulation of osteopontin (*Spp1*) provides additional evidence of impaired bone homeostasis. *Spp1* is a glycoprotein responsible for osteoclast anchoring and enhanced osteoblastic differentiation [41]. *Ckb* downregulation may also account for impaired osteoclastogenesis seen in bone metastases of breast cancer [7,42]. Downregulation of Cathepsin S (*Ctss*) also suggests further disruption in osteoblast and osteoclast differentiation with modulation in bone microarchitecture formation [43]. As in the soft callus, there was a detectable elevation in the expression of muscle specific markers such as *Tnnc2*, *Mylpf*, *Myh4*, *Tnnt3*, *Acta1*, *Eno3*, *Myl1*, *Ckm*, *Actn3*, and *Pvalb*. These may represent alterations in muscle function in MDA-MB-231. However, a more likely explanation is that muscle-specific markers were included in the interzone tissue detection spots of the MDA-MB-231 callus whereas the interzone region of interest of the wild-type was drawn without inclusion of the surrounding muscle. One of the current limitations of spatial transcriptomics is the lack of single-cell resolution. While deconvolution techniques exist, each tissue detection spot (~55 um in diameter for Visium CytAssist) can include up to 10 individual cells, which may lead to heterogeneity within tissue detection spots that complicates comparison of genetic expression profiles between regions of interest. Thus, conclusions drawn from this preliminary report should be considered carefully in the wider context of the available literature.

### 4.4. Downstream Analysis of Biomarker Genes

The high multiplex of the Visium CytAssist spatial transcriptomics platform makes the interpretation of differential gene lists difficult. Pathway analysis is one validated mechanism of analyzing high multiplex data streams and can identify canonical pathways of interest and predicted upstream regulators [44,45]. Pathway analysis identified cancer as a top canonical pathway for all three regions, suggesting that differential gene expression between the regions of interest reflect the transcriptomic changes induced by the presence of MDA-MB-231 cells. Given that human cells were used, the transcriptomic changes detected are reflective of the mouse only. *TGFBI* and *TP53* were both identified as upstream regulators of interest in both the soft callus and interzone. *TP53* is well characterized as a key player in metastatic cancer, especially in breast cancer [46]. The role of *TGFBI* in bone and cancer is bidirectional. *TGFBI* may have a tumor suppressor or tumor promoter role depending on the stage of tumor progression [47].

### 4.5. Spatial Transcriptomics Shows Temporal Patterning

The fracture callus is a dynamic region with a temporal and spatial architecture. Comparison of the one-week and two-week MDA-MB-231 interzones was used to illustrate temporality. The bridging region of the one-week sample was taken as the interzone. Fibronectin 1 (*Fn1*) was down-regulated in the one-week sample compared to the two-week sample, reflective of hematoma resolution [48]. A total of 145 differentially expressed genes in the interzone demonstrated temporal patterning changes during fracture healing from one to two weeks. Early wound healing genes (*Actb*, *Postn*, *Fn1*, *Tnc*) showed higher expression in the one-week interzone as soft tissue repair and hematoma formation resolved. The diversity and variance between the two temporally different samples may suggest that the fibrous interzone is a rapidly evolving area of fracture repair.

### 4.6. Biological Replicates

The inclusion and usage of biological replicates with spatial transcriptomics is a controversial topic that has not yet been clearly outlined. With differing techniques in slide preparation, RNA quality, and transcriptomic technique, a universal cutoff may not be applicable. Williams et al. suggested the cutoff DV200 of > 50% [49]. However, the Visium CytAssist spatial transcriptomics platform (10× Genomics) recommends a cutoff of >30% as sufficient for reproducibility. The DV200 index assesses a percentage of RNA fragments greater than 200 nucleotides, which has shown greater correlation with RNA quality than the traditional RNA integrity number [50]. Previous literature studies involving this platform have ranged from one to four replicates per group [51,52,53,54,55]. However, power analysis is challenging given that spatial features are unknown as well as the high complexity of spatial data [56]. In this study, we report the use of one sample per group as a preliminary application and examination. Additional replicates may be required as the technology evolves.

Limitations of this study include the small sample size, limited time points, and restriction to a single breast cancer cell lineage. High-throughput techniques such as spatial transcriptomics are more susceptible to producing false positive discoveries. Additional confirmation with other sequencing techniques is required for further supporting evidence. Data in this study and spatial transcriptomics in general do not generate conclusive evidence given subjectivity in data interpretation and filtering criteria. Confirmatory studies should be performed to elucidate significant expression changes. The murine model of the callus may differ slightly across mouse types. NOD/scid-IL2Rγcnull have shown small differences in bone properties, although at the early stages of fracture healing used in this study, no appreciable differences in callus content were found in comparison to BALB/C [57]. This is a preliminary study to demonstrate the application of spatial transcriptomics in bone.

## 5. Conclusions

We provide a preliminary report of the application of spatial transcriptomics for the spatial and temporal characterization of femur fracture callus gene expression patterns in normal and pathological fractures. The technique successfully identified literature validated hard callus (*Dmp1*, *Sost*) and soft callus (*Acan*, *Col2a1*) genes. The technique also identifies novel genes within the bridging interzone, supporting its role as a key zone of fracture healing regulation. Finally, we provide evidence that the highly metastatic, triple-negative breast cancer cell line (MDA-MB-231) induces significant disruption of bone homeostasis in all three regions of interest. Additional focused studies are necessary to characterize targetable biomarker genes for analysis. Spatial transcriptomics may be a powerful, hypothesis-driving tool in orthopedics research.

## Data Availability

The data presented in this study are available on request from the corresponding author.

## Supplementary Materials

The following supporting information can be downloaded at: https://www.mdpi.com/article/10.3390/cells13060522/s1. Table S1. Sample quality. Table S2. Unsupervised global gene clustering. Top expressed genes of each cluster sorted by *p*-value for the two-week wild-type sample. Table S3. Unsupervised global gene clustering. Top expressed genes of each cluster sorted by *p*-value for the two-week MDA-MB-231 sample. Table S4. Unsupervised global gene clustering. Top expressed genes of each cluster sorted by *p*-value for the one-week MDA-MB-231 sample. Table S5. Complete differential gene list of the comparison of the two-week MDA-MB-231 hard callus and the two-week wild-type hard callus. Gene list calculated by ANOVA and filtered by *p*-value < 0.05, false discovery rate (FDR) < 0.01, and fold change at least from −1 to 1. Table S6. Complete differential gene list of the comparison of the two-week MDA-MB-231 soft callus and the two-week wild-type soft callus. Gene list calculated by ANOVA and filtered by *p*-value < 0.05, false discovery rate (FDR) < 0.01, and fold change at least from −1 to 1. Table S7. Complete differential gene list of the comparison of the two-week MDA-MB-231 interzone and the two-week wild-type interzone. Gene list calculated by ANOVA and filtered by *p*-value < 0.05, false discovery rate (FDR) < 0.01, and fold change at least from −1 to 1.
