# Supplementary material for: Unique Spatial Transcriptomic Profiling of the Murine Femoral Fracture Callus: A Preliminary Report
Source: Cells. 2024 Mar 16;13(6):522. doi: 10.3390/cells13060522 (PMC10969736; doi:10.3390/cells13060522)
Supplement: Supplementary file 1 [file cells-13-00522-s001.zip › cells-2881591-supplementary.pdf]

Supplementary Table S1. Sample quality.

Sample	Number of Reads	Valid UMIs	Reads Mapped to Probe Set	Fraction Reads in Spots under Tissue	Mean Reads per Spot	Median UMI Counts per Spot	Median Genes per Spot	Genes Detected
1 Week MDA231	263,182,368	100.0%	98.6%	98.9%	47,089	855	447	15,533
2 Week MDA231	320,224,301	100.0%	98.3%	97.7%	49,077	651	347	16,281
2 Week Wild-Type	254,517,220	100.0%	98.5%	98.6%	46,293	676	392	15,823

Supplementary Table S2. Unsupervised global gene clustering. Top expressed genes of each cluster sorted by p-value for the two-week wild-type sample.

Top features	Cluster 1	p-value	fold change	Cluster 2	p-value	fold change	Cluster 3	p-value	fold change	Cluster 4	p-value	fold change	Cluster 5	p-value	fold change
1	Bglap	8.86E-104	13.04	Col2a1	7.19E-44	3.88	Timp2	1.08E-69	19.77	Tmcc2	4.51E-51	1.72	Il1rn	2.79E-110	93.25
2	Col1a1	1.84E-68	3.71	F13a1	5.94E-41	28.49	Igfbp7	7.87E-49	5.58	Ighm	9.86E-49	1.50	Fth1	1.62E-97	48.03
3	Gja1	2.84E-67	11.79	Comp	3.41E-37	3.43	Igfbp5	1.65E-47	16.74	Ank1	1.67E-37	1.95	Ly6e	1.72E-65	16.22
4	Acp5	6.81E-63	12.33	Mgp	2.17E-36	4.96	Mmp2	1.49E-43	6.38	Acta1	7.89E-34	15.61	Lgals3	2.00E-63	73.36
5	Col1a2	7.99E-55	2.37	Cst3	1.82E-33	22.71	Comp	3.19E-39	3.86	Lcn2	3.58E-32	1.57	Ctsl	1.44E-49	11.82
6	Id3	4.34E-47	7.25	Alpl	4.44E-26	4.11	Bgn	7.66E-39	9.76	Myh4	1.87E-28	20.71	Psap	3.70E-43	14.96
7	Tnc	1.06E-45	11.06	Ibsp	4.89E-25	6.78	Mxra8	4.31E-38	8.89	Ckm	5.46E-28	16.02	Crip1	1.07E-42	15.51
8	Ctsk	9.29E-41	6.86	Phospho1	4.16E-23	9.35	Tgfbi	3.40E-33	4.63	Tnnc2	2.52E-27	12.12	Tspo	4.25E-40	8.45
9	Mmp9	1.99E-38	6.70	Chil1	3.66E-19	10.70	Apoe	1.44E-32	12.91	Arhgdib	3.26E-27	1.77	Bst2	4.36E-39	34.77
10	Ckb	2.26E-37	7.06	Ptp4a1	3.14E-18	4.79	Col6a3	2.81E-27	3.47	Bpgm	7.66E-26	1.52	Nfkbia	2.20E-38	33.36
11	Sparc	1.64E-34	6.07	Fn1	1.47E-17	1.96	Hspg2	2.46E-26	3.54	Atp2a1	9.29E-25	19.63	Ctss	6.44E-37	11.55
12	Col11a2	1.94E-34	2.83	Pth1r	1.24E-15	5.48	Col6a2	5.76E-26	2.85	Mylpf	1.10E-24	7.77	Actb	1.49E-35	3.82
13	Cfh	1.71E-32	8.58	Gpc1	2.49E-13	3.78	Lmna	4.23E-25	5.02	Ccna2	3.99E-24	1.82	Ctsd	2.39E-35	16.62
14	Nrp2	7.88E-30	6.22	Chst12	1.27E-09	2.06	Cebpd	6.64E-25	4.89	Cybb	7.37E-24	1.83	Txn1	1.13E-32	11.03

15	Satb2	6.02E-29	5.91	Timp3	3.00E-08	4.56	Crip1	8.15E-25	2.85	Tnnt3	3.87E-23	13.14	S100a6	1.04E-31	4.83
16	Col11a1	2.12E-26	5.55	Gpx3	3.19E-08	2.18	Lsp1	3.24E-23	2.16	Fam111a	2.08E-22	1.51	Vim	3.60E-31	12.24
17	Csf1r	4.43E-26	13.51	Ckap4	4.36E-07	3.16	Ctsl	4.93E-23	3.55	Pygl	2.10E-22	1.64	B2m	5.78E-30	3.24
18	Serpinf1	5.36E-23	4.31	Lman1	1.25E-06	2.98	Ski	1.65E-22	6.08	Pf4	5.02E-22	1.59	Tapbp	6.48E-30	10.90
19	Lifr	1.25E-21	4.82	Pgam1	5.05E-06	3.25	Gpx3	3.34E-22	3.02	Pvalb	8.67E-21	20.72	Cd44	1.42E-29	9.67
20	Car3	8.88E-21	1.61	Sdc4	1.60E-05	3.07	Lgals1	4.22E-21	4.69	Pdcd4	4.69E-19	1.63	Ifitm3	4.52E-29	11.51
21	Jdp2	1.49E-19	5.67	Fxyd1	2.36E-05	1.78	Fn1	1.05E-20	2.38	Ttn	1.94E-16	7.66	Tgm2	2.07E-28	13.54
22	Slc4a2	2.30E-18	9.58	Rcn3	3.48E-05	2.54	Col5a1	4.07E-20	2.76	Prkcb	7.56E-16	1.52	Ctsb	2.25E-27	11.10
23	Hexa	5.06E-17	3.78	Clint1	2.04E-04	2.30	Clu	6.22E-20	7.43	Eno3	1.42E-15	10.99	Esd	6.66E-27	16.05
24	Hexb	9.40E-17	11.77	Scp2	6.28E-04	1.74	Mgp	7.51E-20	2.44	Ptprc	3.00E-15	2.31	Cyba	9.37E-26	2.32
25	Prex1	3.38E-14	5.41	Rpn1	6.67E-04	1.91	Cd81	3.50E-19	8.25	Cd74	3.23E-15	1.65	Tagln2	4.35E-24	3.55
26	Arsb	1.84E-09	8.05	Npm1	7.17E-04	1.56	Vim	4.80E-19	2.79	Hnrnpul1	8.18E-15	1.54	Ctsz	2.05E-23	9.61
27	Bmp1	3.71E-09	1.94	Mif	0.00105478	1.77	Lum	1.04E-18	3.74	Alox5ap	1.15E-13	1.64	Fcer1g	2.79E-23	2.75
28	Tns3	3.82E-09	2.84	Hnrnpab	0.00124852	1.66	Csrp1	1.44E-17	4.75	Rad23a	1.56E-13	2.21	Junb	7.83E-23	10.11

29	Atp6v1e1	5.33E-09	3.23	Serpinh1	0.00135534	2.20	Mtch1	7.65E-17	5.82	Anxa1	1.71E-12	1.65	C3	1.35E-22	12.61
30	Plxnd1	6.13E-08	2.93	Hspg2	0.00279993	1.50	Col3a1	7.92E-17	1.98	Ybx3	7.10E-12	2.10	Itgb2	2.25E-22	4.31
31	Tmem176b	8.83E-08	2.53	Rpn2	0.00662751	1.71	Col2a1	1.88E-14	2.23	Gda	1.07E-11	1.55	Cd300lf	2.49E-22	3.69
32	Tcirg1	1.02E-07	2.82	Dynlrb1	0.0099381	2.48	Jund	1.94E-14	4.26	Myl1	1.26E-10	3.08	Gpx1	4.74E-22	1.54
33	Atp5g3	1.37E-07	2.03	Fgfr1	0.0126394	1.65	Ctss	8.45E-14	4.08	Pfkm	2.84E-10	4.90	Tgfbi	7.20E-22	5.71
34	Pck2	1.67E-07	1.55	Vcl	0.02459664	2.02	Col4a1	2.84E-12	3.05	Gpi1	3.94E-10	2.22	Selplg	1.56E-21	2.30
35	Serpine2	2.47E-07	1.68	Eprs	0.03077761	3.14	App	2.98E-12	3.71	Tpm1	7.61E-10	5.62	Slpi	1.04E-20	4.44
36	Atp6v0c	4.14E-07	3.17	Ndufb6	0.03398001	1.58	Rgs2	4.95E-12	7.62	Tpm2	3.38E-08	3.03	Cd36	1.02E-19	4.90
37	Atp6v1f	8.50E-07	1.77	Selenoh	0.03479249	1.67	Gas6	1.27E-11	1.89	Serinc3	1.20E-07	2.49	Srgn	2.57E-19	2.15
38	Mdh1	1.47E-06	1.74	2310022B05Rik	0.04213511	2.41	Slc6a6	2.39E-11	3.42	Pygm	1.44E-07	3.81	Plac8	4.90E-19	1.85
39	Itgb3	1.65E-06	1.98	Brd3	0.04853603	1.77	S100a6	3.35E-11	1.69	Myh1	3.20E-07	6.98	Ahnak	6.16E-19	11.53

40	Edem1	2.76E-06	1.76	Cuedc2	0.0575265 8	1.67	Ctsb	1.98E-10	2.34	Hnrnpk	1.52E-06	1.86	Spp1	1.77E-18	8.18
41	Cyc1	7.75E-06	1.63	Ebna1bp2	0.0596127 7	1.70	Col4a3bp	4.88E-10	4.74	Bsg	1.67E-06	1.64	Stx11	5.32E-18	2.73
42	Pcolce	8.52E-06	1.64	Rabac1	0.0833374 2	1.95	Mt1	5.44E-10	2.07	Myo1f	2.30E-06	1.75	Cd68	8.80E-18	8.03
43	Atp1a1	1.10E-05	1.57	Mapk3	0.1013885 1	1.82	Plvap	9.48E-10	3.96	Flna	2.71E-06	1.88	Rap1b	2.18E-17	2.42
44	Ahcyl1	1.86E-05	2.40	Srp9	0.1131749 1	1.63	Thbs1	1.02E-09	2.30	Car3	1.29E-05	4.53	H2-K1	3.85E-17	12.86
45	Fgfr1	2.01E-05	1.58	Kdelr2	0.1173278 2	1.59	Cxcl14	1.12E-09	2.61	Cyb5a	1.43E-05	1.74	Hk3	4.15E-17	2.06
46	Mmp13	3.47E-05	2.19	Slpi	0.1317769 9	2.69	Col5a2	2.82E-09	1.92	Gsr	2.18E-05	1.79	Tap2	4.99E-17	2.91
47	Tmem176a	9.15E-05	1.69	Swi5	0.1954486 6	1.53	B4galt1	3.75E-09	2.04	Mybpc2	4.15E-05	2.69	Sqstm1	1.22E-16	8.09
48	Laptm5	9.74E-05	1.59	Mbnl1	0.2237195 5	1.74	Il6st	5.60E-09	7.63	Aldoa	4.48E-05	4.19	Lmna	4.12E-16	5.32
49	Ctsa	1.23E-04	2.16	Hpcal1	0.2341025 5	1.57	Slc40a1	8.17E-09	2.14	Lamtor4	4.87E-05	1.68	S100a4	4.59E-16	16.64
50	Kctd12	1.29E-04	1.88	Slc25a5	0.2443854 8	1.67	Ubc	4.20E-08	1.73	Ezr	1.59E-04	1.66	Fcgr3	4.77E-16	2.25

51	Cd63	2.57E-04	1.59	Mapk14	0.31518537	1.63	Anxa5	5.77E-08	2.93	H2-Eb1	2.59E-04	1.55	Tnfrsf1a	6.00E-16	7.80
52	Atp6v0b	2.60E-04	1.55	Ppp3ca	0.33521562	2.16	Zyx	9.23E-08	1.53	B2m	5.97E-04	1.68	Plec	7.08E-16	4.33
53	Cox6a1	3.68E-04	1.73	Psmb4	0.35291492	1.56	Ptms	1.09E-07	2.51	Cdca4	0.0010339	1.61	Smox	8.08E-16	5.12
54	Selenop	4.70E-04	1.52	Serpine2	0.44169416	1.78	Nfe2l1	3.37E-07	3.85	H2afz	0.00114851	1.62	Cfl1	1.22E-15	2.73
55	Sdhb	6.98E-04	1.62	Casp3	0.53213763	1.78	Mbtps1	4.52E-07	1.85	Cnp	0.00144558	1.56	Nadk	1.60E-15	5.91
56	Srsf9	0.00277984	1.54	Snf8	0.53670668	2.40	Wwp2	6.05E-07	3.04	Dnmt1	0.00290595	4.03	Lgals9	1.67E-15	3.08
57	Parvb	0.00346971	2.38	Casq1	0.53823538	2.79	Upf1	1.28E-06	2.93	Srsf11	0.004017	8.98	Grn	3.56E-15	6.62
58	Gusb	0.0066241	2.92	Usf2	0.59093624	1.60	Prkacb	1.63E-06	5.07	Tram1	0.00427583	1.54	H3f3b	5.48E-15	2.30
59	Mgat4b	0.01243391	2.35	Calm2	0.65207622	1.59	Laptm4a	1.86E-06	1.54	Atp5a1	0.0054682	1.58	Npc2	6.07E-15	2.95
60	Ak2	0.01848954	1.69	Atp2c1	0.70344008	1.53	Junb	1.86E-06	2.23	Trir	0.00730267	1.86	Aldh3b1	1.00E-14	3.03
61	Sec61a1	0.01896322	1.51	Stx4a	0.74841932	3.41	Fabp4	2.13E-06	2.31	Slc48a1	0.00747575	1.55	Sod2	1.23E-14	9.14

62	Atp6v0e	0.0197409 7	2.08	Xpnpep1	0.7543726 1	1.77	Sdf2	2.39E-06	1.95	Ube2s	0.0089622 1	1.93	Alox5ap	1.42E-14	1.77
63	Scand1	0.0310617 4	1.51	Mt1	0.8254511 5	1.51	Anapc5	2.45E-06	1.65	Ywhaz	0.0099959 3	1.68	Btg1	1.63E-14	3.14
64	Sh3kbp1	0.0343719 5	1.74	Lyn	0.8792745 6	4.14	Orai1	2.52E-06	1.63	Ubr3	0.0177261 6	1.54	Clic1	1.74E-14	3.75
65	Map2k3	0.0494689	1.50	Maf	0.9069210 1	1.89	Ctsd	2.65E-06	2.25	Mpc2	0.0285457 8	1.52	Gna13	1.83E-14	3.83
66	Vapa	0.0808290 5	1.59	Uba2	0.9268809 4	1.54	Eng	2.86E-06	2.55	Gtf2i	0.0392164 5	2.87	Fxyd5	1.91E-14	5.52
67	Crebzf	0.0890157 7	1.95				Colgalt1	4.10E-06	3.30	Skp1a	0.0396289 2	1.78	Litaf	2.74E-14	3.98
68	Oxct1	0.1454443 6	2.03				Anxa6	5.24E-06	2.41	Oaz1	0.1846284 9	1.87	Msn	4.08E-14	4.11
69	Naa50	0.2539117 7	1.78				Sptbn1	6.79E-06	1.67	Myl12a	0.2835835 1	2.37	Msrb1	4.35E-14	1.81
70	Dcaf12	0.2718793 7	1.62				Tsc22d3	8.36E-06	1.90	Mgat1	0.3822569 9	1.57	Esyt1	7.28E-14	2.38
71	Bex3	0.3041756 3	2.72				Sdc4	9.29E-06	1.95	Hnrnpm	0.3879031 4	1.74	Ezr	2.53E-13	3.04
72	Actr1a	0.3204810 3	2.66				Cdc42se1	1.73E-05	5.94	Sfpq	0.3982879 3	1.91	Tyrobp	1.54E-12	2.38

73	Tmem131	0.3427333 8	1.64				Myo1c	1.90E-05	1.61	Ifitm3	0.490691	2.45	Cd47	1.84E-12	1.96
74	Cbx1	0.3653231 5	1.56				Cbfb	2.82E-05	2.39	Plec	0.5032512 5	2.11	Btg2	2.82E-12	1.79
75	Prdx4	0.3879852 4	1.89				Ccnl2	3.93E-05	2.20	Numa1	0.5688083 5	2.43	Cd52	3.65E-12	1.65
76	Nisch	0.4063098 2	1.54				Tcp1l2	4.64E-05	1.85	Colgalt1	0.6677135 8	1.58	Mpeg1	3.77E-12	3.09
77	Macf1	0.4573818	1.70				Tmem165	5.00E-05	1.50	Wwp2	0.7419120 4	1.62	Psme1	3.88E-12	2.38
78	Ndufs4	0.4592960 6	1.78				Cat	5.63E-05	1.55	Rer1	0.9895598 9	4.37	Anxa2	4.69E-12	3.27
79	R3hdm4	0.5437674 4	1.53				Ostf1	8.61E-05	1.68				Calm1	8.02E-12	3.71
80	Rgl2	0.5747811 1	2.83				Adipor1	1.02E-04	3.33				Plekho2	1.80E-11	2.50
81	Bclaf1	0.6306403	2.40				Lamp1	2.69E-04	2.58				Mxd1	2.91E-11	2.29
82	Eif4ebp1	0.7505554 2	1.60				Tapbp	2.85E-04	1.69				Myo1f	3.69E-11	2.99
83	Nktr	0.8705181 6	2.30				Fcgr3	2.92E-04	1.71				Grina	4.24E-11	1.91

84	Preb	0.9508834	1.52				Rabac1	2.94E-04	2.49				Sdcbp	6.18E-11	1.91
85							Cct3	3.19E-04	1.86				Apbb1ip	1.45E-10	1.93
86							Prrc2a	3.54E-04	1.69				Myl12a	1.57E-10	4.99
87							Actn1	3.97E-04	3.93				Col6a3	1.58E-10	2.85
88							Traf7	4.55E-04	2.37				Pkm	1.95E-10	2.41
89							Ywhaq	6.14E-04	2.63				Csf3r	2.94E-10	10.33
90							Rnh1	6.35E-04	1.88				Capza2	3.17E-10	3.55
91							Sh3pxd2b	8.82E-04	2.05				Gch1	3.34E-10	1.52
92							Limd1	9.00E-04	1.56				Pfn1	3.67E-10	1.72
93							Plpp3	9.19E-04	2.64				Capg	5.82E-10	3.33
94							Stk25	0.00109552	1.58				Arpc3	1.00E-09	1.99
95							Rp2	0.00127454	3.27				Cxcl14	1.02E-09	7.21
96							Epn1	0.00205416	4.62				Axl	1.57E-09	7.62
97							Slc25a4	0.00208472	2.61				BC0055537	2.53E-09	4.72

98							Sar1a	0.00215542	2.08				Slc14a1	2.56E-09	1.53
99							2-Sep	0.00221913	1.64				Mcl1	2.71E-09	2.12
100							Map7d1	0.00263529	2.07				Igfbp7	3.17E-09	2.32
101							Arf1	0.00277565	1.81				Myo1c	3.27E-09	4.56
102							Tcp1	0.00287701	1.91				Gusb	6.57E-09	2.18
103							Ddx6	0.00304744	1.55				Picalm	8.43E-09	8.90
104							Wdr61	0.00335825	1.54				Chmp4b	9.47E-09	7.55
105							Fis1	0.0036403	1.53				Psmb9	1.00E-08	2.23
106							Eif3h	0.0042976	2.01				Tnfaip2	1.18E-08	5.93
107							Gak	0.00463339	2.19				Atp6ap2	1.91E-08	2.18
108							Rgcc	0.00521665	4.05				Prdx5	1.93E-08	2.02
109							Dctn3	0.0053379	1.73				Mdm2	2.27E-08	5.51

110							Itgb1	0.00622057	1.59				Rnh1	4.52E-08	7.42
111							Msl1	0.00640009	1.55				Apoe	6.05E-08	2.65
112							Ptpra	0.00689473	1.65				Col6a2	7.26E-08	2.09
113							Rab11a	0.0070705	1.90				Clec2d	9.90E-08	1.64
114							Anxa2	0.00782928	1.55				Glipr2	1.03E-07	4.34
115							Klf13	0.00916025	1.50				Col4a1	1.11E-07	2.60
116							Cdk4	0.00924191	1.67				Cd9	1.48E-07	3.48
117							Tmed3	0.00974691	3.98				Ehbp1l1	1.52E-07	2.19
118							Lamp2	0.01019436	1.75				Gda	1.66E-07	2.11
119							Hnrnpf	0.0107336	2.93				Glud1	1.68E-07	3.16
120							Ralbp1	0.0111088	2.67				Stard7	1.73E-07	2.72
121							Tnpo1	0.01142405	1.52				Zyx	1.75E-07	1.87

122							Hsd17b11	0.01158994	1.80				Ucp2	1.82E-07	2.15
123							Snx5	0.01206499	1.64				Efhd2	2.21E-07	5.63
124							Cenpb	0.01387302	1.97				Klf13	2.74E-07	2.11
125							2-Mar	0.0158815	1.76				Arhgdia	3.13E-07	1.53
126							Pdcd6ip	0.01639648	1.79				Cltc	4.40E-07	1.58
127							Srsf6	0.0177723	1.64				Cotl1	4.97E-07	2.37
128							Atp2a2	0.02058673	1.51				Trpv2	5.33E-07	1.69
129							Ppic	0.02087683	1.52				Tln1	5.69E-07	1.88
130							Sri	0.02775008	1.72				Col3a1	5.94E-07	1.79
131							Mycbp2	0.02848889	1.51				Rhog	9.88E-07	1.70
132							Myh1	0.02997833	2.07				Plpp3	1.07E-06	3.31

133							Dad1	0.03312259	2.18				Akna	1.16E-06	1.60
134							Ube4b	0.03456542	2.16				Atp6v0c	1.26E-06	1.80
135							Lrp10	0.03756939	2.16				Anxa5	1.31E-06	4.91
136							Cdc45	0.03995488	1.55				Gdi2	1.64E-06	1.91
137							5-Sep	0.04106207	1.78				H2-T23	1.81E-06	1.70
138							Ttn	0.04269249	2.15				Atox1	1.95E-06	3.87
139							Glud1	0.04854621	2.44				Marcksl1	2.22E-06	3.01
140							Egln2	0.05627601	1.86				Slfn2	2.32E-06	1.75
141							Idh2	0.05648264	1.87				Mif	2.97E-06	2.29
142							Pxk	0.05956752	1.92				Cast	3.41E-06	2.13
143							Kif1c	0.06591968	2.85				Dcn	4.50E-06	5.65

144							Psmc3	0.06593177	2.54				Atp6v0e	4.73E-06	2.39
145							Eif4a1	0.0661567	1.76				Fn1	4.87E-06	1.75
146							Ssr3	0.073245	2.06				Diaph1	5.63E-06	1.56
147							Pole4	0.07799701	2.04				Anxa7	5.76E-06	2.19
148							Tmem127	0.08458296	1.55				Sptlc2	5.88E-06	1.96
149							Myl1	0.09627752	2.29				Lrp10	6.05E-06	2.05
150							Ubr4	0.10363702	1.58				Unc93b1	7.17E-06	2.03
151							Papola	0.10618429	1.98				Lpgat1	8.47E-06	2.15
152							Gna12	0.10623771	1.53				Sh3glb1	8.48E-06	2.19
153							Pygm	0.10703873	2.86				Vps26a	1.00E-05	2.05
154							Ilrun	0.11261868	3.88				Lgals1	1.02E-05	2.78

155							Aldoa	0.11445746	2.33				Itgam	1.02E-05	1.71
156							Mprip	0.12387985	2.17				S100a11	1.07E-05	1.70
157							Arpc1b	0.13095064	1.62				Wdr1	1.20E-05	2.20
158							Mknk2	0.16932419	1.81				Nploc4	1.35E-05	2.44
159							Pgp	0.19823583	4.25				Sh3bgrl3	1.40E-05	2.33
160							Mybpc2	0.20204702	3.50				B4galt1	1.42E-05	3.47
161							Actn3	0.20834581	5.34				Actn1	1.65E-05	1.66
162							Laptm5	0.21986876	1.87				Ankfy1	1.95E-05	1.98
163							Get4	0.23515121	1.56				Myh9	1.97E-05	1.58
164							Hnrnph2	0.24415479	1.70				Selenot	2.13E-05	2.04
165							Igsf6	0.27311737	6.28				Capn2	2.16E-05	1.97

166							Atp6ap1	0.29550416	2.16				Pgam1	2.31E-05	1.74
167							Ranbp9	0.30057924	1.58				Gnb2	2.38E-05	1.87
168							Tbrg1	0.32488752	1.71				Usp25	2.43E-05	1.71
169							Psmd2	0.34656055	1.56				Spi1	2.60E-05	2.36
170							Atp5o	0.34920587	2.32				Pim1	2.73E-05	2.19
171							Parp1	0.40550693	1.69				Znrf1	2.79E-05	1.96
172							Pspc1	0.46184109	3.03				Tmem259	3.28E-05	1.87
173							Ndufv2	0.51039103	1.85				Csrp1	3.84E-05	3.33
174							Hsbp1	0.5268459	2.61				Riok3	3.87E-05	1.52
175							Uqcrh	0.56121115	1.76				Emp3	4.90E-05	2.60
176							Mylpf	0.57057994	2.44				Pitpna	5.29E-05	1.85

177							Ino80e	0.7162037	1.83				Hmox1	5.86E-05	10.18
178							Arcn1	0.77744057	1.69				Vdac2	5.90E-05	2.54
179							Pdha1	0.92703454	1.63				Plvap	6.33E-05	3.85
180							St3gal1	0.96040946	1.53				Rab11a	6.45E-05	1.89
181													Rtf2	6.55E-05	1.87
182													Fkbp1a	8.16E-05	2.33
183													Fli1	8.73E-05	2.48
184													Acsl5	8.78E-05	2.14
185													Flot2	9.04E-05	2.77
186													Polr2j	1.24E-04	2.40
187													Specc1	1.24E-04	1.79
188													Nsd2	1.48E-04	1.60
189													Pdcd6ip	1.67E-04	1.81
190													Ppt1	1.83E-04	2.33
191													Actr3	1.87E-04	2.54

192													Adgre5	1.87E-04	1.58
193													Ywhag	1.89E-04	2.48
194													Bag1	1.90E-04	1.77
195													Grb2	1.99E-04	1.94
196													Zc3hav1	2.10E-04	1.91
197													Rab5c	2.30E-04	1.55
198													Dbi	2.33E-04	1.54
199													Eng	2.38E-04	4.64
200													Plxnd1	2.55E-04	1.92
201													Ddx5	2.65E-04	1.65
202													Tbc1d2b	2.65E-04	3.63
203													Canx	2.79E-04	1.89
204													Nap1l4	3.01E-04	2.22
205													Arel1	3.05E-04	2.59
206													Vasp	3.12E-04	1.69
207													Iqgap1	3.25E-04	1.57

208													H2-Eb1	3.28E-04	2.04
209													Ostf1	3.52E-04	1.86
210													Akr1a1	3.65E-04	2.06
211													Ebna1bp2	3.67E-04	2.59
212													Atp6v0b	4.08E-04	1.75
213													Psmb3	4.37E-04	2.01
214													Mif4gd	4.59E-04	4.98
215													Cebpd	4.67E-04	3.07
216													Srrt	4.68E-04	1.58
217													Spsb3	4.71E-04	1.60
218													Cct2	4.72E-04	1.61
219													Pcmt1	4.86E-04	1.62
220													Gls	5.00E-04	1.62
221													Lmbrd1	5.37E-04	2.17
222													Csnk1a1	5.89E-04	1.76
223													Ssna1	6.31E-04	4.09

224													Syngr2	6.33E-04	6.74
225													Mtpn	6.34E-04	2.99
226													Ank	6.60E-04	6.40
227													Lrrfip1	7.03E-04	1.53
228													Hsp90b1	8.11E-04	1.80
229													Hist1h2bc	9.21E-04	2.72
230													Sirpa	0.00101494	1.53
231													Hsd17b11	0.00106597	1.80
232													Bptf	0.0011148	1.79
233													Nfe2l2	0.00124337	1.50
234													Rhoa	0.00141494	4.07
235													Vsir	0.00144377	2.51
236													Psmd4	0.00152369	2.11

237													Ranb p9	0.001 5286	1.62
238													Capn 1	0.001 7260 1	1.77
239													Ago2	0.001 8437 7	1.76
240													Uqcr 11	0.002 0248 9	1.93
241													Irf2b p2	0.002 0902 5	1.84
242													Spop	0.002 3320 4	1.86
243													Psmb 2	0.002 4176 6	2.73
244													Arf6	0.002 5788 3	1.58
245													Sdf2	0.002 6326 7	1.70
246													Map k3	0.002 6520 5	1.71
247													Fbx w11	0.002 7789	1.73

248													Ncf4	0.0030582 8	1.70
249													Cnot8	0.0031530 1	1.72
250													Psmc6	0.0035074 2	1.68
251													Kat6a	0.0035146 8	1.70
252													Fkbp8	0.0042679 4	1.54
253													Col5a1	0.0044391 9	1.54
254													Map3k3	0.004552	5.97
255													Sdha	0.0045670 8	1.86
256													Arpc4	0.0047043 5	2.52
257													Sars	0.0051631 1	1.72
258													Ptpn1	0.0054417 5	1.87

259													Hspg2	0.00565399	1.73
260													Mia2	0.00582356	2.47
261													Cct6a	0.00599163	11.69
262													Capns1	0.00600638	2.03
263													Get4	0.00639538	1.93
264													Tcf25	0.00729464	1.82
265													Htra2	0.00730456	2.19
266													Lypla2	0.00782066	2.12
267													Atp2a2	0.00793253	1.81
268													Ptbp1	0.00813417	1.77
269													Gabarapl2	0.0090322	1.60

270													Hbp1	0.00971061	1.71
271													Med13l	0.00994876	1.79
272													Ogdh	0.01159702	3.08
273													Ppp6r1	0.01255184	2.24
274													Pgk1	0.01271026	3.94
275													Eif2s2	0.01363058	1.70
276													Cyb5a	0.01374907	1.53
277													Tram1	0.01430083	1.77
278													Tcirg1	0.01603171	2.09
279													Tsc22d3	0.01722999	1.97
280													Tmem134	0.01750218	1.52

281													Mbd3	0.0177084	1.52
282													Flna	0.0179425 6	1.67
283													Bcl2l1	0.0181136 4	1.57
284													Psma2	0.0191540 8	3.41
285													Zmat2	0.0195073 7	3.47
286													Traf7	0.0223382 5	1.71
287													Dnajb1	0.0299636 2	1.50
288													Stx16	0.0331499 7	1.51
289													Cops7a	0.0346510 3	2.08
290													Syncrip	0.0357136 1	1.64
291													Tsc22d1	0.0359126 5	5.90

292													Tm9 sf2	0.037 1328 7	2.40
293													Nme 1	0.038 3304 2	1.95
294													Map 7d1	0.038 5154 5	1.52
295													U2af 1	0.039 4074 5	1.75
296													Eif4g 2	0.040 2235 5	1.52
297													Nduf b1-ps	0.042 1714 5	1.53
298													Psen en	0.045 7264 7	2.35
299													Ppp2 ca	0.053 9394 5	3.73
300													Suds 3	0.055 2422 4	1.87
301													Lyst	0.056 8017 4	1.51
302													Cdkn 2c	0.058 3771 5	1.60

303													Ap2a2	0.0584554 6	1.51
304													Pkn1	0.0589965 6	1.63
305													Mob1a	0.0599763 7	4.82
306													Snrpe	0.0645961 5	2.32
307													Hexa	0.0654926 8	1.74
308													Ppp2r5c	0.0667834 4	1.85
309													App	0.0716705 2	1.82
310													Ptpn11	0.0758450 8	1.63
311													Sh3bgrl	0.0786915	1.62
312													Wnk1	0.0793694 6	1.76
313													Ptpn12	0.0804127 1	1.99

314													Szrd1	0.08741365	1.61
315													Pafah1b1	0.08877722	1.55
316													Actr10	0.09141187	2.25
317													Dctn2	0.09417268	1.59
318													Tubb4b	0.12126573	1.65
319													Ptms	0.12276605	1.67
320													Psmb7	0.12676466	1.65
321													Tra2a	0.13122822	2.32
322													Fam168b	0.14141837	1.79
323													Cmas	0.14556908	1.86
324													Ube2b	0.18306319	1.62

325													Pds5a	0.18903589	1.69
326													Nars	0.21012452	1.64
327													Vars	0.21409802	1.65
328													Mtor	0.22299943	1.67
329													Surf4	0.22804354	2.12
330													Orai1	0.23253443	1.58
331													Atp1a1	0.24812516	1.77
332													Vps35	0.25177926	1.65
333													Cpsf6	0.25247076	2.05
334													Sri	0.2798421	1.59
335													Itm2c	0.28633711	1.77

336													Stt3b	0.33243304	1.53
337													Ppp1r8	0.38736926	1.67
338													Atp5c1	0.38880334	2.03
339													Supt5	0.41604737	1.69
340													Eif3l	0.4478203	1.82
341													Ndufb8	0.49153387	2.40
342													Kidins220	0.49567928	2.02
343													Asap1	0.70816818	1.61
344													Mtch1	0.77135836	1.63
345													Ubald2	0.81025844	1.58
346													Uqcrh	0.86513783	1.59

347													Marcks	0.8953677	2.67
348													Ssr3	0.9069953 6	1.83
349													Ppic	0.9306925 9	1.53
350													Ndufa2	0.9786105 5	3.02

Supplementary Table S3. Unsupervised global gene clustering. Top expressed genes of each cluster sorted by p-value for the two-week MDA-MB-231 sample.

Top features	Cluster 1	p-value	fold change	Cluster 2	p-value	fold change	Cluster 3	p-value	fold change
1	Col1a2	4.08E-97	2.89	Col3a1	1.38E-93	5.34	Col2a1	1.26E-177	51.98
2	Col1a1	1.89E-71	2.79	Crip1	1.13E-56	16.42	Col9a1	1.02E-152	39.51
3	Bglap	1.15E-38	11.72	Col6a2	4.58E-56	6.15	Col9a3	1.63E-136	39.80
4	Acp5	1.83E-33	5.47	Tnn	6.68E-55	14.98	Comp	3.28E-97	19.55
5	Dmp1	7.87E-33	5.32	Lmna	1.65E-54	6.10	Col9a2	4.10E-89	16.79
6	Ctsk	6.96E-32	6.78	Acta1	3.53E-49	9.94	Acan	6.10E-76	20.68
7	Col11a2	8.61E-30	2.85	Col6a1	6.56E-49	6.19	Mgp	5.94E-69	13.58
8	Nrp2	3.02E-28	2.54	Vim	6.20E-48	7.84	Cst3	6.63E-29	6.10
9	Gja1	1.85E-25	2.30	Tgfbi	4.16E-47	8.86	Ccdc80	1.63E-21	3.42
10	Id3	6.59E-24	2.27	Ecm1	7.86E-45	12.80	Smoc2	1.74E-17	6.77
11	Ckb	9.08E-22	3.69	Aebp1	1.33E-43	7.23	Cspg4	2.63E-17	2.20
12	Col22a1	3.15E-20	2.41	Thbs2	1.72E-43	6.83	Rbp4	1.50E-16	4.65
13	Mmp9	6.25E-16	1.89	Col6a3	5.37E-43	5.74	Fn1	1.75E-10	2.14
14	Col11a1	1.37E-12	1.90	Col5a1	1.62E-42	3.15	Sdc4	2.68E-08	2.48
15	Ptgis	3.34E-11	1.82	Postn	2.70E-41	5.48	Alpl	3.80E-08	1.73
16	Creb3l1	2.04E-10	2.47	Tnnc2	4.66E-41	7.37	Col16a1	1.21E-07	1.87
17	Phospho1	3.12E-10	2.28	Ckm	4.11E-37	7.31	Gpc1	4.70E-07	2.25
18	Csf1r	1.35E-09	2.89	Mylpf	1.85E-35	6.94	Emilin1	5.46E-07	2.17
19	Cd68	9.40E-08	1.89	Angptl2	1.47E-34	5.08	Igfbp7	1.57E-06	1.89
20	Ibsp	7.26E-07	2.34	Mmp2	1.23E-32	4.37	Fscn1	0.00258535	1.67
21	Hexa	2.63E-06	1.81	S100a6	3.92E-32	4.76	Fibin	0.00853956	1.66
22	Spp1	4.58E-06	2.52	Aldoa	1.53E-31	2.92	Mmp9	0.23071441	1.60
23	Tmem176b	1.95E-05	1.86	Fn1	1.96E-30	2.48	Maged1	0.29991692	1.55
24	Hba-a2	1.93E-04	1.91	Plec	3.54E-28	3.30	Fgfr1	0.34024572	1.58

25	Atp6v0e	0.00199742	1.79	Igfbp7	5.98E-28	2.05	Hnrnpab	0.35671937	1.69
26	Hbb-bs	0.00260241	1.56	Fstl1	3.13E-27	5.08	Mt1	0.56442893	1.71
27	Mmp13	0.00487388	1.78	Timp2	1.39E-26	4.30			
28				Tnnt3	2.20E-26	4.61			
29				Ybx3	1.77E-25	3.25			
30				Ahnak	3.54E-25	3.23			
31				Myh4	8.57E-25	5.55			
32				Lgals1	1.24E-24	2.75			
33				Igfbp5	1.53E-24	4.93			
34				Fth1	2.70E-24	4.20			
35				Loxl2	2.72E-22	4.40			
36				Lgals3	1.23E-21	4.67			
37				Bgn	2.44E-19	2.23			
38				Ctsl	3.64E-19	3.49			
39				Emp1	8.20E-19	3.44			
40				Hspg2	2.77E-18	2.25			
41				Tagln2	2.85E-18	2.65			
42				Fibin	5.75E-18	3.20			
43				Txn1	1.29E-17	2.53			
44				Nbl1	1.82E-16	2.15			
45				Lum	3.74E-16	2.23			
46				Antxr1	1.00E-14	2.68			
47				Jund	1.89E-14	2.00			
48				Cthrc1	4.96E-14	2.26			
49				Col4a2	7.92E-14	4.09			
50				Tuba1a	8.52E-14	1.91			
51				Ski	9.86E-14	2.10			
52				Col12a1	1.52E-13	2.33			

53				Cd81	1.85E-13	2.01				
54				Col4a1	6.35E-13	4.04				
55				Pkm	9.44E-13	2.56				
56				Myo1c	2.97E-12	2.49				
57				Cd44	5.09E-12	3.17				
58				Tnfrsf1a	8.69E-12	2.32				
59				Apoe	1.30E-11	3.67				
60				Ywhag	3.48E-11	2.31				
61				Pmepa1	2.59E-10	1.64				
62				Ctsb	1.27E-09	1.84				
63				Col16a1	2.65E-09	1.92				
64				Tpm1	3.04E-09	2.27				
65				Actb	1.01E-08	1.89				
66				Mxra8	1.48E-08	1.90				
67				Psap	2.49E-08	1.96				
68				C1qtnf6	3.11E-08	1.60				
69				Cd47	9.60E-08	2.48				
70				Mtch1	1.43E-07	2.13				
71				Sqstm1	1.75E-06	1.87				
72				Gpi1	3.08E-06	2.01				
73				Rap1b	5.49E-06	2.12				
74				Plvap	8.20E-06	2.10				
75				Dcn	3.28E-05	1.66				
76				Gpx1	3.68E-05	2.22				
77				Tnfaip2	5.18E-05	1.50				
78				Msn	5.46E-05	1.56				
79				Ywhaz	1.14E-04	1.70				
80				Nid2	1.16E-04	1.53				

81				Pdia3	1.31E-04	1.71				
82				Ubc	1.89E-04	1.69				
83				Ctsd	0.00139589	1.51				
84				Hist2h2bb	0.00468357	1.63				
85				Hnrnpm	0.0159233	1.66				
86				Ube2m	0.01888249	1.58				
87				Calm1	0.02370051	1.58				
88				Pgam1	0.03051486	1.56				
89				Ssr3	0.07433097	1.50				
90				Car3	0.75330208	2.05				

Supplementary Table S4. Unsupervised global gene clustering. Top expressed genes of each cluster sorted by p-value for the one-week MDA-MB-231 sample.

Top features	Cluster 1	p-value	fold change	Cluster 2	p-value	fold change
1	Tgfbi	1.99E-18	11.30	Col1a2	5.01E-22	1.77
2	Col6a2	3.80E-18	7.72	Col1a1	1.03E-19	2.63
3	Fn1	2.56E-17	5.36	Mmp9	7.71E-18	4.91
4	Tnn	1.60E-16	14.95	Acp5	4.20E-15	5.22
5	Col3a1	2.49E-16	3.08	Dmp1	5.87E-15	2.20
6	Angptl2	1.48E-12	7.47	Col22a1	2.34E-13	2.18
7	Aebp1	2.26E-12	11.05	Ckb	7.53E-13	8.19
8	Col6a1	1.82E-11	6.14	Car3	2.88E-12	2.99
9	Crip1	6.07E-11	6.41	Gja1	6.98E-12	2.21
10	Thbs2	7.37E-11	8.36	Ctsk	2.53E-11	4.60
11	Col6a3	1.10E-10	3.17	Mmp13	3.19E-10	11.81
12	Lmna	2.71E-10	2.71	Slc4a2	4.40E-08	4.55
13	Postn	4.21E-10	13.52	Nrp2	8.11E-08	1.97
14	Igfbp7	6.72E-10	1.87	Hexa	9.08E-08	2.50
15	Fth1	1.33E-08	2.15	Id3	7.35E-07	1.66
16	Col5a1	1.66E-08	3.67	Gpc1	2.97E-06	1.62
17	Fibin	3.65E-08	3.91	Bglap	4.13E-06	4.51
18	S100a6	4.53E-08	3.11	Ptgis	7.46E-06	3.06
19	Bgn	1.01E-07	2.50	Atp6v1e1	2.48E-05	2.44
20	Pmepa1	1.80E-07	2.69	Sh3pxd2b	6.38E-05	1.71
21	Acan	1.82E-07	11.90	Spp1	1.33E-04	2.32
22	Comp	6.41E-07	13.75	Tnnc2	2.60E-04	2.25
23	Emilin1	8.03E-07	3.40	Cyc1	0.00143806	1.74
24	Col9a3	1.37E-06	15.71	Ctsz	0.00196439	1.88

25	Grb10	2.67E-06	4.08	Cd68	0.00370229	1.88
26	Smoc2	2.69E-06	1.88	Ckm	0.00448134	1.89
27	Pdia3	3.57E-06	2.06	Tmem176b	0.0060072	1.63
28	Igfbp5	1.22E-05	2.20	Creb3l1	0.0063904	1.68
29	Mgp	1.34E-05	6.62	Tnnt3	0.00884771	2.53
30	Mmp2	1.40E-05	1.87	Ctsa	0.00884807	1.60
31	Jund	3.22E-05	2.36	Uqcrc1	0.01471704	1.52
32	Fstl1	4.23E-05	3.58	Myh4	0.01853007	1.91
33	Col9a1	4.73E-05	14.00	Mylpf	0.02335842	1.76
34	Tagln2	5.07E-05	2.32	Ahcyl1	0.04906869	1.51
35	Myo1c	5.56E-05	1.98	Slc39a7	0.06207363	1.85
36	Apoe	5.90E-05	9.98	Atp6v0e	0.11104512	1.51
37	Loxl2	6.33E-05	3.39	Tnc	0.27646613	1.97
38	Timp2	6.77E-05	1.76	Hist4h4	0.49098454	1.64
39	Pkm	8.52E-05	1.89	Slc25a5	0.59083628	2.06
40	Cd81	1.12E-04	1.94	Mt1	0.62433476	1.54
41	Hspg2	1.55E-04	1.79			
42	Hsp90b1	1.79E-04	1.56			
43	Col9a2	2.35E-04	10.28			
44	Tuba1a	3.51E-04	2.44			
45	Ppib	4.21E-04	1.77			
46	Ckap4	4.52E-04	1.56			
47	Surf4	5.01E-04	1.53			
48	Ctsl	5.13E-04	2.50			
49	Sdc4	8.16E-04	2.73			
50	Ddb1	0.00110138	1.63			
51	Thbs1	0.0012533	1.57			
52	Col2a1	0.00125895	8.18			

53	Vim	0.00223164	1.79			
54	Myl12a	0.00282791	1.72			
55	Cthrc1	0.00332182	1.66			
56	Ctsb	0.00384687	3.23			
57	Mtch1	0.00482792	2.05			
58	Rbp4	0.00510327	3.41			
59	Fscn1	0.00574327	1.76			
60	Tmsb4x	0.00628898	2.22			
61	Col12a1	0.00633081	2.23			
62	Sar1a	0.00690348	1.50			
63	Tnfrsf1a	0.0081746	1.63			
64	Lgals1	0.00916693	1.84			
65	Rabac1	0.00998772	1.62			
66	Rer1	0.01183203	1.70			
67	H2afz	0.01385191	1.69			
68	Txn1	0.01447579	1.94			
69	Maged1	0.01690518	1.51			
70	Arhgdia	0.02055006	1.66			
71	Plec	0.0235455	1.99			
72	Ptbp1	0.03015833	1.57			
73	Gpi1	0.03269063	2.08			
74	Cspg4	0.03691878	3.25			
75	Ecm1	0.04542868	1.66			
76	C1qtnf6	0.06759725	2.13			
77	Nbl1	0.06965241	1.92			
78	App	0.10396289	1.57			
79	Akt1	0.16513958	1.53			
80	Sfpq	0.19742708	1.65			

81	Lgals3	0.24769173	1.74			
82	Gnb2	0.51315775	1.62			

Supplementary Table S5. Complete differential gene list of the comparison of the two-week MDA-MB-231 hard callus and the two-week wild-type hard callus. Gene list calculated by ANOVA and filtered by p-value <0.05, false discovery rate (FDR) <0.01, and fold change at least from -1 to 1.

Gene	P-value	FDR step up	Ratio	Fold change	LSMean(MDA-231)	LSMean(WT)
Acta1	1.01E-18	2.92E-16	4.38E-01	0.44	-2.28	2.40
Sem1	8.59E-17	1.24E-14	1.64E+00	1.64	1.64	4.33
Ckm	7.27E-16	7.00E-14	5.57E-01	0.56	-1.80	1.78
Hba-a2	4.64E-14	3.35E-12	2.28E+00	2.28	2.28	3.60
Tnnc2	4.93E-12	2.85E-10	6.58E-01	0.66	-1.52	2.15
Aldoa	8.33E-11	4.01E-09	7.22E-01	0.72	-1.39	2.08
Mylpf	2.51E-10	1.04E-08	7.11E-01	0.71	-1.41	1.46
Pgam2	1.10E-09	3.97E-08	8.03E-01	0.80	-1.25	1.06
Pvalb	4.20E-09	1.35E-07	7.53E-01	0.75	-1.33	1.17
Tnc	3.13E-08	9.03E-07	5.76E-01	0.58	-1.74	4.76
Bglap	3.60E-08	9.47E-07	4.91E-01	0.49	-2.04	16.18
Selenop	2.46E-07	5.92E-06	6.65E-01	0.67	-1.50	2.09
Thbs1	4.08E-07	8.68E-06	6.38E-01	0.64	-1.57	3.36
Col12a1	4.21E-07	8.68E-06	7.27E-01	0.73	-1.38	1.73
Igfbp5	1.35E-06	2.61E-05	7.53E-01	0.75	-1.33	1.43
Eno3	1.67E-06	2.91E-05	7.60E-01	0.76	-1.32	1.33
Tpm1	1.71E-06	2.91E-05	7.52E-01	0.75	-1.33	4.39
Actn3	2.08E-06	3.34E-05	8.38E-01	0.84	-1.19	1.15
Tcap	5.17E-06	7.87E-05	8.48E-01	0.85	-1.18	1.14
Ndufb7	5.85E-06	8.22E-05	7.94E-01	0.79	-1.26	1.52
Col4a2	5.97E-06	8.22E-05	7.27E-01	0.73	-1.38	1.90
Myoz1	8.07E-06	1.06E-04	8.31E-01	0.83	-1.20	1.14
Cthrc1	1.05E-05	1.32E-04	6.99E-01	0.70	-1.43	2.07
Tnnt3	1.16E-05	1.39E-04	7.82E-01	0.78	-1.28	1.49

Myh4	1.29E-05	1.50E-04	7.82E-01	0.78	-1.28	1.87
App	1.84E-05	2.04E-04	7.39E-01	0.74	-1.35	3.21
Mdh1	2.02E-05	2.15E-04	7.50E-01	0.75	-1.33	1.67
Lum	2.10E-05	2.15E-04	7.44E-01	0.74	-1.34	2.16
Atp2a1	2.16E-05	2.15E-04	8.08E-01	0.81	-1.24	1.39
Cyc1	2.51E-05	2.42E-04	7.65E-01	0.77	-1.31	2.00
Mybpc2	3.35E-05	3.09E-04	8.07E-01	0.81	-1.24	1.25
Itm2c	3.42E-05	3.09E-04	7.29E-01	0.73	-1.37	2.21
Col6a2	4.58E-05	4.01E-04	7.39E-01	0.74	-1.35	3.23
Eef1a1	4.71E-05	4.01E-04	6.76E-01	0.68	-1.48	5.43
Maf	6.20E-05	5.12E-04	8.03E-01	0.80	-1.25	1.61
Ubc	7.10E-05	5.70E-04	7.97E-01	0.80	-1.25	1.71
Fn1	1.17E-04	9.12E-04	6.76E-01	0.68	-1.48	4.28
Pygm	1.23E-04	9.33E-04	8.74E-01	0.87	-1.14	1.16
Rap1b	1.44E-04	1.07E-03	8.10E-01	0.81	-1.23	1.83
Myl1	1.55E-04	1.12E-03	8.13E-01	0.81	-1.23	1.27
Tnni2	1.59E-04	1.12E-03	9.00E-01	0.90	-1.11	1.04
Ckb	1.78E-04	1.22E-03	6.71E-01	0.67	-1.49	3.43
Tln1	2.22E-04	1.49E-03	8.24E-01	0.82	-1.21	1.62
Calm1	4.06E-04	2.66E-03	8.26E-01	0.83	-1.21	1.66
Olfml2b	4.19E-04	2.69E-03	7.73E-01	0.77	-1.29	1.89
Arhgdia	4.31E-04	2.70E-03	8.06E-01	0.81	-1.24	1.63
Naca	4.40E-04	2.70E-03	8.05E-01	0.81	-1.24	2.02
Lgals3	4.84E-04	2.91E-03	8.02E-01	0.80	-1.25	1.61
Cox6a2	5.06E-04	2.99E-03	9.22E-01	0.92	-1.08	1.04
Cox5b	5.41E-04	3.13E-03	7.80E-01	0.78	-1.28	2.28
Clta	6.34E-04	3.59E-03	8.14E-01	0.81	-1.23	2.15
Vim	8.29E-04	4.59E-03	7.96E-01	0.80	-1.26	2.37
Atp5b	8.41E-04	4.59E-03	8.23E-01	0.82	-1.22	1.51

Rack1	8.80E-04	4.71E-03	7.43E-01	0.74	-1.35	6.08
Cct3	9.51E-04	4.97E-03	8.51E-01	0.85	-1.18	1.56
Psmb4	9.64E-04	4.97E-03	8.44E-01	0.84	-1.18	1.76
Marcks	9.87E-04	5.00E-03	8.07E-01	0.81	-1.24	1.71
Ckap4	1.03E-03	5.14E-03	7.92E-01	0.79	-1.26	3.09
Mxra8	1.05E-03	5.14E-03	7.95E-01	0.79	-1.26	1.95
Atp6v1e1	1.07E-03	5.17E-03	7.86E-01	0.79	-1.27	2.18
Atp5a1	1.16E-03	5.48E-03	8.17E-01	0.82	-1.22	2.29
Antxr1	1.32E-03	6.09E-03	8.25E-01	0.83	-1.21	1.61
Myh1	1.33E-03	6.09E-03	8.68E-01	0.87	-1.15	1.15
Hspg2	1.37E-03	6.17E-03	7.97E-01	0.80	-1.25	2.29
H3f3b	1.39E-03	6.20E-03	7.91E-01	0.79	-1.26	1.76
Ryr1	1.45E-03	6.35E-03	9.09E-01	0.91	-1.10	1.12
Gnas	1.49E-03	6.44E-03	7.89E-01	0.79	-1.27	4.80
Ybx3	1.66E-03	7.06E-03	8.49E-01	0.85	-1.18	1.47
Psap	1.74E-03	7.31E-03	7.55E-01	0.76	-1.32	3.69
Col3a1	1.82E-03	7.50E-03	7.66E-01	0.77	-1.31	12.52
Akr1a1	1.98E-03	8.05E-03	8.06E-01	0.81	-1.24	2.09

Supplementary Table S6. Complete differential gene list of the comparison of the two-week MDA-MB-231 soft callus and the two-week wild-type soft callus. Gene list calculated by ANOVA and filtered by p-value <0.05, false discovery rate (FDR) <0.01, and fold change at least from -1 to 1.

**Gene**	**P-value**	**FDR step up**	**Ratio**	**Fold change**	**LSMean(MDA-231)**	**LSMean(WT)**
Col12a1	7.10E-09	2.05E-06	4.95E-01	-2.02	1.42	2.86
Gpx3	2.60E-08	3.76E-06	5.67E-01	-1.76	4.36	7.68
Col6a1	6.20E-08	4.93E-06	5.71E-01	-1.75	2.07	3.63
Eef1a1	6.83E-08	4.93E-06	5.25E-01	-1.90	4.13	7.86
Arf4	1.40E-07	8.09E-06	5.93E-01	-1.69	1.56	2.63
H3f3b	6.61E-07	2.86E-05	6.38E-01	-1.57	1.38	2.16
Lum	6.92E-07	2.86E-05	5.60E-01	-1.79	1.69	3.02
Col5a2	1.70E-06	5.60E-05	5.09E-01	-1.97	5.70	11.21
Col5a1	1.74E-06	5.60E-05	4.94E-01	-2.02	4.08	8.27
Mmp2	3.14E-06	9.07E-05	5.66E-01	-1.77	2.19	3.87
Itm2c	4.61E-06	1.21E-04	6.21E-01	-1.61	1.77	2.86
Sparc	5.51E-06	1.33E-04	4.85E-01	-2.06	5.87	12.10
Myh4	1.22E-05	2.60E-04	1.47E+00	1.47	2.71	1.85
Thbs1	1.26E-05	2.60E-04	5.95E-01	-1.68	3.15	5.30
Fkbp8	1.98E-05	3.81E-04	7.09E-01	-1.41	1.58	2.23
Olfml2b	2.71E-05	4.89E-04	6.50E-01	-1.54	1.38	2.12
Tgfb1	3.11E-05	5.10E-04	6.53E-01	-1.53	1.69	2.59
Actb	3.18E-05	5.10E-04	6.44E-01	-1.55	5.55	8.61
Ctsb	3.35E-05	5.10E-04	6.09E-01	-1.64	1.96	3.23
Aebp1	4.40E-05	6.36E-04	6.36E-01	-1.57	1.88	2.96
Tpt1	5.43E-05	7.47E-04	6.21E-01	-1.61	2.43	3.91
Hspg2	6.55E-05	8.60E-04	6.16E-01	-1.62	2.90	4.71
Psap	7.28E-05	9.14E-04	7.08E-01	-1.41	3.00	4.24
Col6a2	7.60E-05	9.15E-04	6.00E-01	-1.67	5.87	9.79

Cox6a1	1.07E-04	1.13E-03	7.24E-01	-1.38	1.55	2.14
Mxra8	1.09E-04	1.13E-03	6.33E-01	-1.58	1.68	2.65
Ptms	1.09E-04	1.13E-03	7.09E-01	-1.41	2.35	3.31
Serpinf1	1.09E-04	1.13E-03	6.79E-01	-1.47	1.46	2.16
Cthrc1	1.27E-04	1.27E-03	6.24E-01	-1.60	1.83	2.94
Ptp4a3	1.55E-04	1.49E-03	8.02E-01	-1.25	1.07	1.34
Ski	1.61E-04	1.50E-03	6.92E-01	-1.45	1.37	1.98
Serf2	1.77E-04	1.60E-03	7.22E-01	-1.38	1.52	2.11
Col6a3	1.92E-04	1.68E-03	6.85E-01	-1.46	3.36	4.90
Dcn	1.97E-04	1.68E-03	6.67E-01	-1.50	1.57	2.36
Tnc	2.17E-04	1.79E-03	5.94E-01	-1.68	2.13	3.59
Tnfrsf1a	2.34E-04	1.88E-03	7.29E-01	-1.37	1.64	2.25
Mylpf	2.49E-04	1.92E-03	1.44E+00	1.44	2.51	1.74
Calm2	2.52E-04	1.92E-03	7.60E-01	-1.32	2.02	2.66
Vim	2.83E-04	2.06E-03	6.53E-01	-1.53	2.82	4.32
Col3a1	2.86E-04	2.06E-03	4.43E-01	-2.26	19.44	43.88
Thbs2	3.02E-04	2.13E-03	5.28E-01	-1.89	1.88	3.56
Ctss	3.17E-04	2.16E-03	6.98E-01	-1.43	1.19	1.71
Ubc	3.21E-04	2.16E-03	7.55E-01	-1.32	1.46	1.94
Rack1	3.75E-04	2.42E-03	6.94E-01	-1.44	4.86	7.00
Myl12a	3.77E-04	2.42E-03	7.42E-01	-1.35	1.94	2.62
Gas6	3.92E-04	2.46E-03	7.55E-01	-1.32	1.14	1.51
Gnai2	4.42E-04	2.72E-03	7.11E-01	-1.41	2.14	3.01
Sem1	4.83E-04	2.91E-03	1.29E+00	1.29	2.92	2.27
Lgals3	5.11E-04	2.99E-03	7.06E-01	-1.42	2.52	3.58
Mgp	5.17E-04	2.99E-03	1.20E+00	1.20	9.44	7.88
Lman1	5.30E-04	3.01E-03	6.78E-01	-1.48	2.21	3.27
Fth1	5.78E-04	3.21E-03	7.24E-01	-1.38	6.77	9.36
Ywhaz	6.37E-04	3.47E-03	7.29E-01	-1.37	1.49	2.04

Pabpc1	6.56E-04	3.51E-03	7.48E-01	-1.34	1.36	1.82
Tln1	7.38E-04	3.87E-03	7.78E-01	-1.29	1.32	1.70
Bmp1	7.52E-04	3.87E-03	6.79E-01	-1.47	1.76	2.60
Arhgdia	7.63E-04	3.87E-03	7.60E-01	-1.32	1.58	2.08
Cd81	8.99E-04	4.42E-03	6.79E-01	-1.47	1.64	2.42
Pcolce	9.03E-04	4.42E-03	6.63E-01	-1.51	1.55	2.34
Ctsl	9.51E-04	4.58E-03	5.79E-01	-1.73	2.55	4.40
Rcn3	9.83E-04	4.66E-03	6.94E-01	-1.44	1.95	2.82
App	1.05E-03	4.89E-03	6.69E-01	-1.49	2.67	3.99
Sept2	1.24E-03	5.66E-03	7.71E-01	-1.30	1.60	2.07
Bgn	1.26E-03	5.66E-03	7.10E-01	-1.41	3.31	4.66
Tgfbi	1.27E-03	5.66E-03	7.45E-01	-1.34	1.89	2.54
Naca	1.35E-03	5.92E-03	7.09E-01	-1.41	1.80	2.53
Fn1	1.45E-03	6.26E-03	7.04E-01	-1.42	12.39	17.61
Serpinh1	1.51E-03	6.35E-03	6.05E-01	-1.65	3.01	4.98
Ssr3	1.52E-03	6.35E-03	7.14E-01	-1.40	1.79	2.50
Cd47	1.54E-03	6.35E-03	7.70E-01	-1.30	1.35	1.75
Sfpq	1.61E-03	6.52E-03	7.54E-01	-1.33	1.61	2.13
Erh	1.63E-03	6.52E-03	7.57E-01	-1.32	1.49	1.97
Akr1a1	1.65E-03	6.52E-03	7.78E-01	-1.29	1.68	2.16
Surf4	1.78E-03	6.89E-03	7.32E-01	-1.37	1.77	2.42
Ctsa	1.79E-03	6.89E-03	7.88E-01	-1.27	1.39	1.77
Id3	2.03E-03	7.62E-03	6.85E-01	-1.46	1.77	2.59
Gnas	2.04E-03	7.62E-03	6.75E-01	-1.48	3.45	5.12
Mdh1	2.06E-03	7.62E-03	7.91E-01	-1.26	1.27	1.61
Ckap4	2.39E-03	8.71E-03	6.98E-01	-1.43	2.57	3.68
S100a6	2.41E-03	8.71E-03	7.91E-01	-1.26	1.57	1.99
Lgals1	2.54E-03	9.08E-03	7.25E-01	-1.38	2.10	2.89
Slc25a5	2.65E-03	9.34E-03	7.42E-01	-1.35	1.85	2.49

Laptm4a	2.83E-03	9.69E-03	7.40E-01	-1.35	2.60	3.51
Ppib	2.83E-03	9.69E-03	7.65E-01	-1.31	1.79	2.34
Atp2a1	2.85E-03	9.69E-03	1.26E+00	1.26	1.94	1.53
Cnn2	2.97E-03	9.97E-03	7.24E-01	-1.38	1.57	2.17

Supplementary Table S7. Complete differential gene list of the comparison of the two-week MDA-MB-231 interzone and the two-week wild-type interzone. Gene list calculated by ANOVA and filtered by p-value <0.05, false discovery rate (FDR) <0.01, and fold change at least from -1 to 1.

**Gene**	**P-value**	**FDR step up**	**Ratio**	**Fold change**	**LSMean(MDA-231)**	**LSMean(WT)**
Ctsl	3.93E-15	7.34E-13	1.79E-01	-5.59	4.32	24.14
Ctsd	5.08E-15	7.34E-13	3.16E-01	-3.17	1.79	5.67
Spp1	1.05E-11	1.01E-09	2.91E-01	-3.44	1.47	5.06
Cd68	1.84E-11	1.33E-09	3.74E-01	-2.67	1.38	3.67
Atp6v0e	2.37E-11	1.37E-09	4.95E-01	-2.02	2.11	4.27
B2m	4.63E-10	2.23E-08	4.45E-01	-2.25	1.63	3.65
Ctsb	7.06E-10	2.69E-08	3.66E-01	-2.73	3.81	10.41
Actb	7.45E-10	2.69E-08	5.04E-01	-1.98	10.04	19.92
Acp5	1.22E-09	3.93E-08	3.43E-01	-2.92	1.82	5.31
Apoe	2.83E-09	8.19E-08	3.16E-01	-3.16	3.01	9.53
Ckb	1.30E-08	3.35E-07	4.83E-01	-2.07	1.22	2.53
Ctss	1.39E-08	3.35E-07	3.57E-01	-2.80	2.39	6.69
Psap	8.60E-08	1.86E-06	4.10E-01	-2.44	5.00	12.18
Ctsz	8.99E-08	1.86E-06	4.69E-01	-2.13	1.88	4.00
Tnnc2	1.98E-07	3.81E-06	1.80E+00	1.80	7.43	4.12
Mylpf	3.57E-07	6.44E-06	1.79E+00	1.79	3.88	2.16
Cyba	6.18E-07	1.05E-05	5.09E-01	-1.97	1.89	3.71
Col2a1	6.62E-07	1.06E-05	2.14E-01	-4.68	13.78	64.45
Mmp9	7.19E-07	1.09E-05	4.30E-01	-2.32	1.89	4.39
Itm2b	9.29E-07	1.34E-05	5.89E-01	-1.70	3.47	5.90
Tgm2	1.15E-06	1.58E-05	5.71E-01	-1.75	1.67	2.92
Calm1	1.42E-06	1.87E-05	5.66E-01	-1.77	1.58	2.80
Eef2	1.51E-06	1.90E-05	6.16E-01	-1.62	2.92	4.73
Acta1	1.63E-06	1.96E-05	1.63E+00	1.63	12.49	7.65

Tnnt3	2.63E-06	2.98E-05	1.64E+00	1.64	3.32	2.02
Cst3	2.68E-06	2.98E-05	6.04E-01	-1.66	3.74	6.18
Grn	3.33E-06	3.56E-05	4.96E-01	-2.02	2.08	4.20
Thbs4	3.62E-06	3.73E-05	2.21E+00	2.21	4.15	1.88
Gusb	5.31E-06	5.30E-05	5.65E-01	-1.77	1.83	3.24
Gnai2	9.98E-06	9.62E-05	6.48E-01	-1.54	2.81	4.33
Mmp13	1.21E-05	1.13E-04	5.78E-01	-1.73	1.40	2.43
Tln1	1.36E-05	1.23E-04	6.50E-01	-1.54	1.47	2.27
Cdkn1a	1.58E-05	1.38E-04	5.83E-01	-1.72	1.82	3.12
Hspa5	1.91E-05	1.63E-04	6.41E-01	-1.56	2.47	3.86
Tpt1	1.98E-05	1.64E-04	6.48E-01	-1.54	3.58	5.53
Lgals3	2.09E-05	1.68E-04	4.22E-01	-2.37	3.74	8.86
Ctsa	2.36E-05	1.85E-04	5.97E-01	-1.68	1.87	3.14
Sqstm1	2.49E-05	1.90E-04	6.54E-01	-1.53	2.39	3.65
Tmsb4x	2.96E-05	2.16E-04	6.26E-01	-1.60	3.50	5.59
Sdcbp	2.99E-05	2.16E-04	6.09E-01	-1.64	1.54	2.53
H3f3b	3.44E-05	2.43E-04	6.25E-01	-1.60	2.01	3.22
Fth1	5.23E-05	3.60E-04	4.32E-01	-2.31	11.89	27.49
Myh4	5.49E-05	3.69E-04	1.75E+00	1.75	4.36	2.49
Ctsk	5.93E-05	3.90E-04	4.89E-01	-2.05	2.79	5.71
Ckm	7.86E-05	5.05E-04	1.37E+00	1.37	6.46	4.71
Igfbp7	8.20E-05	5.15E-04	6.36E-01	-1.57	8.76	13.78
Ctnnb1	1.05E-04	6.46E-04	6.93E-01	-1.44	2.33	3.37
Akr1a1	1.10E-04	6.62E-04	6.32E-01	-1.58	2.17	3.43
Ubc	1.19E-04	6.73E-04	6.52E-01	-1.53	1.92	2.94
Laptm4a	1.22E-04	6.73E-04	6.68E-01	-1.50	3.04	4.55
App	1.22E-04	6.73E-04	6.68E-01	-1.50	3.58	5.37
Gpx1	1.23E-04	6.73E-04	6.26E-01	-1.60	1.76	2.82
Eef1a1	1.23E-04	6.73E-04	6.34E-01	-1.58	5.81	9.16

Clta	1.42E-04	7.62E-04	6.76E-01	-1.48	2.18	3.22
Cox5b	1.60E-04	8.33E-04	6.68E-01	-1.50	2.22	3.33
Gpc1	1.61E-04	8.33E-04	6.41E-01	-1.56	2.68	4.18
Alpl	1.91E-04	9.70E-04	6.05E-01	-1.65	1.56	2.57
Cfl1	2.06E-04	1.03E-03	6.65E-01	-1.50	3.03	4.55
Rack1	2.41E-04	1.18E-03	7.29E-01	-1.37	6.10	8.37
Ibsp	2.73E-04	1.32E-03	7.06E-01	-1.42	1.15	1.63
Myl1	3.58E-04	1.70E-03	1.39E+00	1.39	2.40	1.73
Ywhaz	5.65E-04	2.63E-03	7.27E-01	-1.38	2.18	3.00
Eif4b	6.36E-04	2.92E-03	7.05E-01	-1.42	1.62	2.31
Ddx5	9.56E-04	4.32E-03	6.97E-01	-1.43	1.72	2.47
Atp6v1e1	1.20E-03	5.36E-03	6.92E-01	-1.45	1.74	2.51
Sdc4	1.28E-03	5.40E-03	7.06E-01	-1.42	1.96	2.78
Hbb-bs	1.29E-03	5.40E-03	1.95E+00	1.95	2.43	1.24
Pvalb	1.29E-03	5.40E-03	1.33E+00	1.33	2.39	1.80
Gja1	1.29E-03	5.40E-03	7.19E-01	-1.39	2.10	2.92
Cnn2	1.75E-03	7.22E-03	7.37E-01	-1.36	1.82	2.47
Hba-a2	1.77E-03	7.22E-03	1.81E+00	1.81	2.22	1.22
Marcks	1.82E-03	7.25E-03	7.00E-01	-1.43	1.51	2.16
Tm9sf2	1.83E-03	7.25E-03	7.23E-01	-1.38	1.65	2.29
Arhgdia	1.86E-03	7.27E-03	7.67E-01	-1.30	1.97	2.57
Gpx3	2.05E-03	7.83E-03	7.13E-01	-1.40	5.25	7.37
Hnrnpa2b1	2.06E-03	7.83E-03	7.49E-01	-1.34	2.14	2.86
Gas6	2.14E-03	8.05E-03	7.25E-01	-1.38	1.57	2.16
Eno3	2.19E-03	8.09E-03	1.42E+00	1.42	3.13	2.20
Actn3	2.24E-03	8.09E-03	1.34E+00	1.34	1.89	1.41
Slc25a5	2.24E-03	8.09E-03	7.09E-01	-1.41	2.06	2.90
Car3	2.33E-03	8.31E-03	1.61E+00	1.61	3.46	2.14
Eif4h	2.36E-03	8.33E-03	7.76E-01	-1.29	2.03	2.61

Gnas	2.47E-03	8.59E-03	7.54E-01	-1.33	4.68	6.20
Itm2c	2.50E-03	8.59E-03	7.36E-01	-1.36	2.21	3.00
Ssr3	2.57E-03	8.63E-03	7.07E-01	-1.42	1.89	2.67
Hsp90b1	2.57E-03	8.63E-03	7.65E-01	-1.31	4.03	5.27
Myl12a	2.60E-03	8.63E-03	7.26E-01	-1.38	2.65	3.65
Nadk	2.68E-03	8.72E-03	6.69E-01	-1.49	1.68	2.51
Mgp	2.68E-03	8.72E-03	4.91E-01	-2.04	2.69	5.49
BC005537	2.76E-03	8.86E-03	7.10E-01	-1.41	2.01	2.84
Tnfaip2	2.83E-03	8.98E-03	7.14E-01	-1.40	2.06	2.88
Sept2	3.13E-03	9.82E-03	7.59E-01	-1.32	2.14	2.82
